# Association of Prenatal Exposure to Benzodiazepines and Z-Hypnotics With Risk of Attention-Deficit/Hyperactivity Disorder in Childhood

**DOI:** 10.1001/jamanetworkopen.2022.46889

**Published:** 2022-12-15

**Authors:** Lene Maria Sundbakk, Jon Michael Gran, Mollie E. Wood, Marte Handal, Svetlana Skurtveit, Hedvig Nordeng

**Affiliations:** 1 PharmacoEpidemiology and Drug Safety Research Group, Department of Pharmacy, and PharmaTox Strategic Initiative, Faculty of Mathematics and Natural Sciences, University of Oslo, Oslo, Norway; 2 Oslo Centre for Biostatistics and Epidemiology, Department of Biostatistics, University of Oslo, Oslo, Norway; 3 Department of Epidemiology, Gillings School of Global Public Health, The University of North Carolina at Chapel Hill, Chapel Hill; 4 Department of Chronic Diseases, Norwegian Institute of Public Health, Oslo, Norway; 5 Department of Mental Disorders, Norwegian Institute of Public Health, Oslo, Norway; 6 Department of Child Health and Development, Norwegian Institute of Public Health, Oslo, Norway

## Abstract

**Importance:**

Evidence is limited regarding the safety of prenatal benzodiazepine and z-hypnotic exposure and its association with long-term neurodevelopment in childhood.

**Objective:**

To quantify the associations of the timing and number of intervals of prenatal exposure to benzodiazepines and/or z-hypnotics with the risk of attention-deficit/hyperactivity disorder (ADHD) in childhood.

**Design, Setting, and Participants:**

This cohort study used data from the 1999 to 2008 population-based Norwegian Mother, Father and Child Cohort Study, which are linked to the Medical Birth Registry of Norway, Norwegian Patient Registry, and Norwegian Prescription Database. Two populations of participants were created: a full sample and a mental health sample. The full sample included mothers and their live-born singletons, whereas the mental health sample was restricted to offspring of mothers who reported anxiety, depression, or sleeping problems during pregnancy or 6 months before pregnancy. Data were analyzed from September 2021 to February 2022.

**Exposures:**

Maternal self-report of benzodiazepine and/or z-hypnotic use during pregnancy was grouped into early pregnancy exposure and middle and/or late pregnancy exposure for analysis of the association with timing of exposure, and number of 4-week intervals of exposure was classified (single [1] vs multiple [≥2]) for analysis of the association with number of exposed intervals.

**Main Outcome and Measures:**

The outcome was ADHD, defined as time to ADHD diagnosis or filled prescription for ADHD medication. To control for confounding, inverse probability of treatment–weighted Cox proportional hazards regression models were used. Hazard ratios and 95% CIs were estimated. The weights were derived from propensity score modeling of the probability of benzodiazepine and/or z-hypnotic exposure as a function of potential confounders between the exposure and the outcome, including maternal symptoms of depression and anxiety.

**Results:**

The full sample comprised 82 201 pregnancies, and the mental health sample included 19 585 pregnancies. In total, 681 offspring (0.8%) in the full sample and 468 offspring (2.4%) in the mental health sample were prenatally exposed to benzodiazepines and/or z-hypnotics. After weighting, exposure in early (hazard ratio, 0.74; 95% CI, 0.39-1.94) and middle and/or late (hazard ratio, 0.76; 95% CI, 0.35-1.61) pregnancy was not associated with increased risk of childhood ADHD. There was no evidence of substantial association between the number of exposed intervals during pregnancy and childhood ADHD.

**Conclusions and Relevance:**

Results of this study suggest that there may be no increased risk of childhood ADHD associated with prenatal exposure to benzodiazepines and/or z-hypnotics, regardless of timing of exposure and number of exposed intervals. However, these findings should be interpreted with caution due to low study power.

## Key Points

**Question:**

Is prenatal exposure to benzodiazepines and/or z-hypnotics associated with an increased risk of attention-deficit/hyperactivity disorder (ADHD) in childhood, and do timing of exposure and the number of exposed intervals play a role in this risk?

**Findings:**

In this cohort study of 82 201 pregnancies, no association was found between childhood ADHD and prenatal exposure to benzodiazepines and/or z-hypnotics by timing of exposure or number of exposed intervals.

**Meaning:**

Findings from this study may be reassuring for pregnant individuals in need of benzodiazepines and z-hypnotics, but these findings need to be interpreted with caution due to low study power.

## Teaser

This cohort study assesses the association of maternal benzodiazepine and z-hypnotic use during pregnancy with attention-deficit/hyperactivity disorder (ADHD) among offspring.

## Introduction

Anxiety affects up to 15% of pregnant individuals worldwide^1^ and may require pharmacological treatment with anxiolytic agents, such as benzodiazepines.^2^ Z-hypnotics are benzodiazepine-related drugs that are mainly prescribed to treat mild insomnia.^3^ Between 1% and 4% of individuals are prescribed benzodiazepines and/or z-hypnotics during pregnancy^4,5,6,7^ for a variety of indications.^8,9^ Benzodiazepines and z-hypnotics modulate the γ-amino butyric acid receptor, the principal inhibitory neurotransmitter in the central nervous system,^10^ and the medications cross the placenta and the fetal blood-brain barrier. Consequently, it is biologically plausible that benzodiazepines and z-hypnotics could affect fetal neurodevelopment.^11,12^ Evidence from animal models suggests a true association of benzodiazepines and/or z-hypnotics with neurodevelopment,^13,14,15^ but studies of the developmental trajectories of children with prenatal exposure to benzodiazepines and/or z-hypnotics remain few.^16,17,18^ Attention-deficit/hyperactivity disorder (ADHD) is one of the most common neurodevelopmental disorders^19,20^ that is characterized by inattention, hyperactivity, and impulsivity.^21^ The global prevalence of childhood ADHD is between 2% and 7%,^22^ and the estimated median age at first ADHD diagnosis in European children is between 6.2 and 18.1 years.^19^ To our knowledge, no previous study has examined the association of prenatal exposure to benzodiazepine and/or z-hypnotics with childhood ADHD. Findings from previous studies on childhood behavioral disorders after prenatal exposure to benzodiazepines and/or z-hypnotics are reassuring.^23,24,25^

Benzodiazepines and z-hypnotics are often used intermittently; thus, any time-varying effect of prenatal exposure is important to guide clinical decisions. Confounding factors might change during pregnancy because of the drug exposure or the pregnancy itself. Thus, it is of interest to investigate whether the timing of exposure or the number of exposed intervals introduces different long-term risks in the offspring. The objective of this study was to quantify the associations of the timing and number of intervals of prenatal exposure to benzodiazepines and/or z-hypnotics with the risk of ADHD in childhood.

## Methods

### Data Sources

This large, prospective cohort study was based on the population-based Norwegian Mother, Father and Child Cohort Study (MoBa) linked to the Medical Birth Registry of Norway (MBRN), Norwegian Patient Registry (NPR), and Norwegian Prescription Database (NorPD) via the personal identification numbers of participants. The establishment of MoBa and initial data collection were previously based on a license from the Norwegian Data Protection Authority Agency and received approval from the Regional Committees for Medical and Health Research Ethics. The MoBa cohort is regulated by the Norwegian Health Registry Act. Written informed consent was obtained from all individuals before participation in the MoBa. The current study was approved by the Regional Committees for Medical and Health Research Ethics, region South East. We followed the Strengthening the Reporting of Observational Studies in Epidemiology (STROBE) reporting guideline.

The MoBa is a population-based pregnancy cohort study conducted by the Norwegian Institute of Public Health.^26^ Participants were recruited throughout Norway from 1999 to 2008. Forty-one percent of pregnant individuals in Norway consented to participate in the MoBa. The MoBa cohort includes approximately 114 500 children, 95 200 mothers, and 75 200 fathers. This current study used information from MoBa questionnaire 1 (sent out in gestational weeks 15-17), questionnaire 3 (sent out in gestational week 30), and questionnaire 4 (sent out 6 months post partum) and was based on version 9 of the quality-assured data files, which were released for research in 2016.^27^

The MBRN is a national health registry that contains information on all births in Norway since 1967.^28^ The NPR contains individual-level information on hospital admission and specialist health care from 2008, including date of admission, date of discharge, and primary and secondary diagnoses.^29^ Diagnoses are coded using the *International Statistical Classification of Diseases and Related Health Problems, Tenth Revision* (*ICD-10*). The NorPD contains information about all prescription drugs that have been dispensed to individuals in ambulatory care facilities since 2004.^30^

### Study Populations

**Figure 1.**
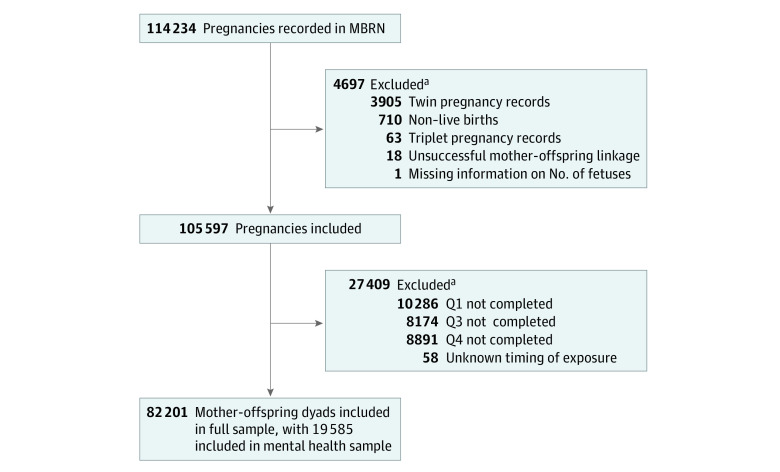
Flowchart of the Final Study Populations MBRN indicates Medical Birth Registry of Norway, and Q indicates questionnaire. ^a^Conditions of exclusion can overlap.

We defined 2 study populations: a full sample and a mental health sample. The full sample included all mother-offspring dyads in which the pregnant individuals completed the MoBa questionnaires 1, 3, and 4. Only live-born singletons were included. To limit confounding by indication, we restricted the full sample to mother-offspring dyads in which the mothers reported in the questionnaires an underlying indication for treatment with benzodiazepines and/or z-hypnotics (anxiety, depression, or sleeping problems) 6 months before pregnancy or during pregnancy, creating the mental health sample (eTable 1 in Supplement 1). This mental health sample emulated the design of a hypothetical randomized clinical trial.^31^ A flowchart of the study population selection is presented in Figure 1.

### Exposure

Benzodiazepine and z-hypnotic exposure information was available from the MoBa questionnaires. Pregnant individuals could report the name of the medication taken for specific symptoms or illnesses they had experienced. The timing of use during pregnancy was reported in 4-week intervals (gestational weeks 0-4, 5-8, and so on). All medications were coded according to the Anatomical Therapeutic Chemical (ATC) groups.^32^ Benzodiazepines were classified as ATC groups N05BA (benzodiazepine-anxiolytics; diazepam, oxazepam, and alprazolam), N05CD (benzodiazepine-hypnotics; nitrazepam, flunitrazepam, and midazolam), and N03AE01 (clonazepam). Z-hypnotics were classified as ATC group N05CF (zopiclone and zolpidem).

Exposure was divided into 2 time windows: early (gestational weeks 0-16) and middle and/or late (gestational weeks 17-28 and/or 29 to delivery). An offspring was classified as exposed in a specific time window if the mother reported use of benzodiazepines and/or z-hypnotics within that time window. We defined number of exposed intervals according to whether the mother reported use in a single 4-week interval or multiple (≥2) 4-week intervals, using the group of those offspring with mothers who reported use in a single 4-week interval as the reference group. We also defined an ever (yes or no) exposure group during pregnancy. The comparison group consisted of offspring with mothers who did not use benzodiazepine and/or z-hypnotics during pregnancy (unexposed).

### Outcome

The outcome was ADHD, defined as time to ADHD diagnosis recorded in the NPR (ie, *ICD-10* code F90: hyperkinetic disorders [F90.0, F90.1, F90.8, or F90.9]) and/or filled ADHD prescription medication (ATC codes N06BA01, N06BA02, N06BA04, N06BA09, and N06BA12) registered in the NorPD. The *ICD-10* code F90 requires the combination of both inattentive and hyperactive symptoms. The validity of ADHD diagnoses has not been investigated for Norwegian registries. A Danish study, set in a similar health care system as in Norway, showed a positive predictive value of 0.87 for registered ADHD diagnosis.^33^ For offspring with no outcome of interest during the study period, follow-up time was available only by year and not by month. Their follow-up time was defined by subtracting the birth year from 2016.5 (eg, offspring born in 1999 with no ADHD outcome would have a follow-up time of 17.5 years). For offspring who experienced the outcome during the study, exact follow-up time was available. We had no information about dates of emigration or death.

### Covariates

Potential confounders were identified through the literature and directed acyclic graphs^34,35^ (eFigures 1 and 2 in Supplement 1). Baseline and time-varying covariates were obtained from the MoBa questionnaires, MBRN, and NorPD. Baseline covariates included maternal age, marital status, parity, prepregnancy body mass index, educational level, gross yearly income, planned pregnancy, folic acid supplementation, smoking habit, alcohol use, illicit drug use, lifetime history of major depression,^36^ sleeping problems, adverse life events, obstetric comorbidity index,^37^ and ADHD medication use. Time-varying covariates included symptoms of depression and anxiety during pregnancy (measured by a 5-item short version of the Hopkins Symptoms Checklist^38^) and comedication use during pregnancy (nonsteroidal anti-inflammatory drugs [NSAIDs], opioids, paracetamol, antidepressants, antipsychotics, or antiepileptics). Additional baseline covariates (paternal characteristics and maternal ADHD traits) were considered in sensitivity analyses. The eMethods in Supplement 1 provides further information.

### Statistical Analysis

We estimated the hazard of ADHD associated with benzodiazepine and/or z-hypnotic exposure using Cox proportional hazards regression models. Follow-up of offspring started at the date of birth and continued until the date of the event (the first ADHD diagnosis or the first ADHD prescription medication filled) or end of the study period (December 31, 2016), whichever occurred first. We used the age of the offspring as the time scale and a quadratic term for birth year to address left-truncation for the offspring born before 2004. We used several approaches to understand associations of the outcome with both timing and number of exposed intervals. We performed analyses in both the full sample and the mental health sample. We conducted unadjusted analyses to obtain crude hazard ratios (HRs) for all exposure groups, with 95% CIs to report precision.

To account for time-varying confounding, we fit marginal structural models with 2 time points using stabilized inverse probability of treatment weights (IPTWs).^39,40^ We estimated the probability of benzodiazepine and/or z-hypnotic treatment using logistic regression models (separately for early and middle and/or late pregnancy exposure) that were conditional on baseline confounders, history of the time-varying confounders, and treatment until the current time point. The resulting 2 IPTWs were multiplied to obtain a final weight.^39^ For the analysis of benzodiazepine and/or z-hypnotic exposure as ever in pregnancy and by number of exposed intervals, we estimated the probability of benzodiazepine and/or z-hypnotic treatment as ever (yes or no) and in the interval windows (single vs multiple [≥2] 4-week intervals) using logistic regression models that were conditional on baseline covariates and baseline values of the time-varying covariates. The IPTWs were generated using R, package ipw (R Foundation for Statistical Computing).^41^ To assess covariate balance between exposed and unexposed groups in the weighted sample, we calculated standardized mean and proportion differences. A difference of less than 0.1 was considered negligible.^42^ Covariates that remained imbalanced after weighting were added to the outcome model. For all analyses, the stabilized IPTWs were used in Cox proportional hazards regression models to obtain weighted HRs with 95% CIs and robust SEs to account for clustering.

Of the included pregnancies, 22.7% pregnant individuals had incomplete data on at least 1 variable (alcohol intake during pregnancy [ 8.2%] and/or maternal depressive or anxiety symptoms during pregnancy [3.2%]). Assuming that data were missing at random, we imputed missing values using multiple imputation by chained equations.^43,44,45^ The imputation procedure included exposure, baseline hazard, and auxiliary variables (eg, maternal age and illnesses, parity, co-medication, and risk factors for the outcome). We created 20 imputed data sets, with a maximum of 10 iterations. The analysis was performed in each imputed data set to estimate the treatment effect, and then estimates from each imputed data set were combined to obtain an overall pooled estimate.^46^ The eMethods in Supplement 1 provides more information.

We performed 13 preplanned subgroup and sensitivity analyses to assess the robustness of the findings to bias from misclassification and confounding. Among those analyses, we restricted the sample to mother-offspring dyads in which the mother used benzodiazepines and/or z-hypnotics during the 6 months before pregnancy and either continued or discontinued use during pregnancy. Furthermore, we conducted analyses to adjust for additional maternal and paternal factors and complete case analyses to compare against estimates from the imputed data. Probabilistic bias analysis was used to evaluate the impact of bias due to misclassification of the outcome and/or exposure.^47,48^ To check whether the results were sensitive to violations of the proportional hazards assumption, we conducted Cox proportional hazards regression models to obtain period-specific HRs (split at offspring age of 9.53 years) and pooled logistic regression models to estimate survival curves that were standardized over baseline covariates.^49^ All sensitivity analyses are described in the eMethods in Supplement 1.

All statistical analyses were performed with R, version 4.0.3.^50^ Data were analyzed from September 2021 to February 2022.

## Results

The full sample comprised 82 201 pregnancies, of which 681 offspring (0.8%) were exposed to benzodiazepine and/or z-hypnotics during gestation. The most common exposures were benzodiazepine-anxiolytics (332 [0.4%]) and z-hypnotics (255 [0.3%]), with 25 mothers using both. More pregnant individuals were exposed in early pregnancy (435 [0.5%]) than in middle and/or late pregnancy (374 [0.5%]) (eTable 2 in Supplement 1), and use of these drugs in a single 4-week interval during pregnancy was more common than use in multiple 4-week intervals (436 [0.5%] vs 245 [0.3%]) (eTable 3 in Supplement 1). The mental health sample included 19 585 pregnancies, of which 468 offspring (2.4%) were prenatally exposed to benzodiazepines and/or z-hypnotics (eTable 2 in Supplement 1).

Baseline characteristics of the samples are presented in Table 1. Compared with the unexposed group, the exposed group was more likely to smoke, drink alcohol, and use illicit drugs (Table 1) as well as use more concomitant medications during early pregnancy, including NSAIDs (4064 of 81 520 [5.0%] vs 74 of 681 [10.9%]), antidepressants (682 [0.8%] vs 92 [13.5%]), and opioids (996 [1.2%] vs 63 [9.3%]) (eTable 4 in Supplement 1). The exposed group also had a higher prevalence of specific health conditions, including sleeping problems (294 [43.2%] vs 12 738 [15.6%]) and symptoms of anxiety and depression (mean [SD] *z* score 0.9 [1.7] vs 0 [1.0]) compared with the unexposed group. There was satisfactory balance of covariates between the exposure groups after weighting (eFigures 3 and 4 in Supplement 1), except for symptoms of anxiety and depression, which were added to the final outcome models. Characteristics of the weights are shown in eTable 5 in Supplement 1.

**Table 1.** Characteristics of Pregnancies by Exposure Status

Characteristic	Individuals by benzodiazepine and/or z-hypnotic exposure, No. (%)			
	Full sample (N = 82 201)		Mental health sample (n = 19 585)	
	Exposed	Unexposed	Exposed	Unexposed
**Maternal characteristics**				
No. of participants	681 (0.8)	81 520 (99.2)	468 (2.4)	19 117 (97.6)
Age, mean (SD), y	31.2 (4.8)	30.2 (4.5)	31.2 (4.7)	30.3 (4.8)
Married or cohabiting	616 (90.5)	78 639 (96.5)	415 (88.7)	18 115 (94.8)
Primiparous	356 (52.3)	44 158 (54.2)	245 (52.4)	9882 (51.7)
Prepregnancy BMI, mean (SD)	23.7 (4.2)	24.0 (4.2)	24.1 (4.4)	24.1 (4.4)
Missing data	13 (1.9)	1983 (2.4)	9 (1.9)	521 (2.7)
College or university educational level^a^	438 (64.3)	53 272 (65.3)	281 (60.0)	11 567 (60.5)
Missing data	2 (0.3)	344 (0.4)	1 (0.2)	89 (0.5)
Smoking during pregnancy	112 (16.4)	6076 (7.5)	82 (17.5)	1814 (9.5)
Missing data	4 (0.6)	1068 (1.3)	2 (0.4)	184 (1.0)
Alcohol intake during pregnancy	155 (22.8)	9451 (11.6)	110 (23.5)	2404 (12.6)
Missing data	64 (9.4)	6689 (8.2)	46 (9.8)	1585 (8.3)
Gross yearly income^b^				
Average	512 (75.2)	62 292 (76.4)	356 (76.1)	14 185 (74.2)
Low	71 (10.4)	7795 (9.6)	48 (10.3)	2358 (12.3)
High	76 (11.2)	8836 (10.8)	46 (9.8)	1981 (10.4)
Missing data	22 (3.2)	2597 (3.2)	18 (3.8)	593 (3.1)
Planned pregnancy	488 (71.7)	66 818 (82.0)	335 (71.6)	14 937 (78.1)
Missing data	4 (0.6)	115 (0.1)	3 (0.6)	32 (0.2)
Folic acid supplementation^c^	401 (58.9)	49 259 (60.4)	274 (58.5)	11 610 (60.7)
Illicit drug use^d^	33 (4.8)	472 (0.6)	25 (5.3)	207 (1.1)
Sleeping problems	294 (43.2)	12 738 (15.6)	294 (62.8)	12 738 (66.6)
Lifetime history of major depression	134 (19.7)	4682 (5.7)	120 (25.6)	2665 (13.9)
Missing data	20 (2.9)	1996 (2.4)	14 (3.0)	470 (2.5)
Depressive or anxiety symptoms during pregnancy at wk 17, mean (SD) *z* score^e^	0.9 (1.7)	0 (1.0)	0.5 (1.3)	0 (1.0)
Missing data	32 (4.7)	2596 (3.2)	23 (4.9)	763 (4.0)
Adverse life event				
No	154 (22.6)	32 914 (40.4)	73 (15.6)	5239 (27.4)
At least 1: not painful	139 (20.4)	20 179 (24.8)	99 (21.2)	4789 (25.1)
At least 1: painful or very painful	388 (57.0)	28 427 (34.8)	296 (63.2)	9089 (47.5)
Co-medication use anytime during pregnancy				
NSAIDs	98 (14.4)	5039 (6.2)	72 (15.4)	1513 (7.9)
Opioids	97 (14.2)	1636 (2.0)	63 (13.5)	577 (3.0)
Paracetamol	444 (65.2)	36 737 (45.1)	304 (65.0)	9679 (50.6)
Antidepressants	111 (16.3)	781 (1.0)	109 (23.3	740 (3.9)
Antipsychotics	44 (6.5)	624 (0.8)	40 (8.5)	243 (1.3)
Antiepileptics^f^	12 (1.8)	264 (0.3)	10 (2.1)	90 (0.5)
Obstetric comorbidity index, mean (SD)^g^	0.8 (1.2)	0.5 (1.0)	0.8 (1.2)	0.6 (1.0)
Missing data	64 (9.4)	6689 (8.2)	46 (9.8)	1585 (8.3)
ADHD prescriptions^h^	25 (3.7)	721 (0.9)	20 (4.3)	366 (1.9)
ADHD symptom level, mean (SD)^i^	1.3 (0.6)	1.1 (0.6)	1.3 (0.6)	1.2 (0.6)
Missing data	254 (37.3)	29 476 (36.2)	175 (37.4)	7195 (37.6)
Year of childbirth^j^				
1999-2001	37 (5.5)	4398 (5.4)	26 (5.5)	895 (4.7)
2002-2004	244 (35.8)	27 661 (33.9)	166 (35.5)	6155 (32.2)
2005-2007	291 (42.7)	37 077 (45.5)	204 (43.6)	9029 (47.2)
2008-2009	109 (16.0)	12 384 (15.2)	72 (15.4)	3038 (15.9)
**Paternal characteristics**				
College or university educational level^a^	310 (45.5)	40 155 (49.3)	260 (55.6)	8913 (46.6)
Missing data	11 (1.6)	770 (0.9)	10 (2.1)	210 (1.1)
ADHD symptom level, mean (SD)^i^	1.4 (0.6)	1.4 (0.5)	1.4 (0.6)	1.4 (0.5)
Missing data	462 (67.8)	54 723 (67.1)	324 (69.2)	12 624 (66.0)
Depressive or anxiety symptoms during pregnancy, mean (SD) *z* score^e^	0.3 (1.3)	0 (1.0)	0.4 (1.4)	0.1 (1.2)
Missing data	192 (28.2)	20 339 (24.9)	134 (28.6)	4787 (25.0)
Lifetime history of major depression	86 (12.6)	6120 (7.5)	65 (13.9)	1959 (10.2)
Missing data	179 (26.3)	20 176 (24.7)	124 (26.5)	4713 (24.7)
Age, y				
<25	26 (3.8)	3556 (4.4)	20 (4.3)	1065 (5.6)
25-39	533 (78.3)	69 791 (85.6)	363 (77.6)	15 914 (83.2)
40-49	110 (16.1)	7477 (9.2)	76 (16.2)	1967 (10.3)
>49	4 (0.6)	514 (0.6)	3 (0.6)	119 (0.6)
Missing data	8 (1.2)	182 (0.2)	6 (1.3)	52 (0.3)

Abbreviations: ADHD, attention-deficit/hyperactivity disorder; BMI, body mass index (calculated as weight in kilograms divided by height in meters squared); NSAIDs, nonsteroidal anti-inflammatory drugs.

^a^ Included completed or ongoing education.

^b^ Average income indicates approximately between US $14 214 and $47 759, low income indicates $14 214 or less, and high income indicates $46 801 or higher.

^c^ Folic acid supplementation before pregnancy or during pregnancy.

^d^ Illicit drug use during pregnancy or the last month before pregnancy.

^e^ Presence of depressive or anxiety symptoms measured with the 5-item short version of the Hopkins Symptoms Checklist.

^f^ Antiepileptics did not include clonazepam.

^g^ The obstetric comorbidity index was adapted from Bateman et al.^37^ The following variables available in the Medical Birth Registry of Norway or Norwegian Mother, Father and Child Cohort Study were used: asthma, cardiac disease, kidney disease, congenital heart disease, drug abuse, placenta previa, diabetes, chronic hypertension, gestational hypertension, previous cesarean delivery, lupus, age, and severe preeclampsia. The variables were weighted, consistent with Bateman et al.^37^

^h^ Indicates filled prescription for ADHD medication.

^i^ Measured by the World Health Organization Adult ADHD Self-Report Scale screener, as a sum score ranging from 0 (indicating no ADHD symptoms) to 4 (indicating moderate to severe symptoms).

^j^ Categorized in this table only for illustrative purposes.

### ADHD Outcome

Offspring were followed up for a mean (SD) period of 11.3 (2.2) years in the full sample and 11.2 (2.2) years in the mental health sample. A total of 2.7% of offspring in the full sample had ADHD (diagnosis: 2.3%; filled prescription: 2.4%); the outcome was more common in the mental health sample (total: 3.9%; ADHD diagnosis: 3.3%; filled prescription: 3.4%). In the full sample, the mean (SD) age at first diagnosis was 8.5 (2.0) years, and the mean (SD) age at first filled prescription was 9.2 (2.1) years (eFigure 5 in Supplement 1). In the mental health sample, the mean (SD) age at first diagnosis was 8.2 (1.9) years, and the mean (SD) age at first filled prescription was 8.9 (2.0) years.

### Main Analyses

**Figure 2.**
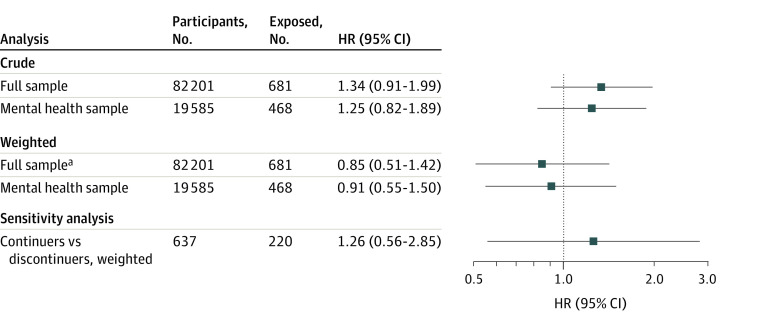
Associations of Exposure to Benzodiazepines and/or Z-Hypnotics Anytime During Pregnancy With Childhood Attention-Deficit/Hyperactivity Disorder in the Full and Mental Health Samples and for the Sensitivity Analysis Comparing Continuers With Discontinuers HR indicates hazard ratio. ^a^The model for the full sample was additionally adjusted for depressive or anxiety symptoms (using the 5-item short version of the Hopkins Symptoms Checklist) because the weights could not balance that covariate.

Crude analyses of the full sample suggested a higher risk of ADHD associated with ever exposure to benzodiazepine and/or z-hypnotics during pregnancy (Figure 2; eFigure 6 in Supplement 1) and early in the pregnancy (Table 2). After weighting, we observed no increased risk of ADHD associated with ever exposure (weighted HR, 0.85; 95% CI, 0.51-1.42) early exposure (weighted HR, 0.74; 95% CI, 0.39-1.94), and middle and/or late exposure (weighted HR, 0.76; 95% CI, 0.35-1.61) during pregnancy (Figure 2; eFigure 7 in Supplement 1; Table 2). Crude analyses of the mental health sample suggested a higher risk of ADHD associated with ever exposure to benzodiazepine and/or z-hypnotics during pregnancy (Figure 2) and early in the pregnancy (Table 2). After weighting, we observed no increased risk of ADHD associated with ever exposure during pregnancy (weighted HR, 0.91; 95% CI, 0.55-1.50) (Figure 2; eFigure 8 in Supplement 1) or in any time window during pregnancy (early exposure: weighted HR, 1.08 [95% CI, 0.61-1.94]; middle and/or late exposure: weighted HR, 0.72 [95% CI, 0.34-1.56]) (Table 2).

**Table 2.** Associations of Prenatal Benzodiazepine and/or Z-Hypnotic Exposure With Childhood ADHD in the Full and Mental Health Samples

	No. of offspring	Events, No.	IR per 1000 person-years	HR (95% CI)	
				Crude	Weighted^a^
**Full sample (N = 82 201)**					
Timing					
Exposed, early pregnancy	435	19	3.9	1.55 (0.96-2.52)	0.74 (0.39-1.94)
Unexposed, early pregnancy	81 766	2236	2.4	1 [Reference]	1 [Reference]
Exposed, middle and/or late pregnancy	374	13	3.1	1.10 (0.62-1.98)	0.76 (0.35-1.61)
Unexposed, middle and/or late pregnancy	81 827	2242	2.4	1 [Reference]	1 [Reference]
No. of intervals^b^					
Exposed, single 4-wk interval	436	13	2.6	1 [Reference]	1 [Reference]
Exposed, multiple (≥2) 4-wk intervals	245	12	4.4	1.67 (0.73-3.84)	1.48 (0.63-3.45)
**Mental health sample (n = 19 585)**					
Timing					
Exposed, early pregnancy	321	18	5.0	1.41 (0.84-2.36)	1.08 (0.61-1.94)
Unexposed, early pregnancy	19 264	741	3.4	1 [Reference]	1 [Reference]
Exposed, middle and/or late pregnancy	261	12	4.1	1.02 (0.54-1.90)	0.72 (0.34-1.56)
Unexposed, middle and/or late pregnancy	19 324	747	3.5	1 [Reference]	1 [Reference]
No. of intervals^b^					
Exposed, single 4-wk interval	268	11	3.6	1 [Reference]	1 [Reference]
Exposed, multiple (≥2) 4-wk intervals	200	12	5.3	1.48 (0.62-3.54)	1.32 (0.55-3.17)

Abbreviations: ADHD, attention-deficit/hyperactivity disorder; HR, hazard ratio, IR, incidence rate.

^a^ Inverse probability of treatment weights. Models for timing of exposure were additionally adjusted for depressive or anxiety symptoms (using the 5-item short version of the Hopkins Symptoms Checklist) because the weights could not balance that covariate.

^b^ The number of weeks did not imply that the pregnant individuals used the medication continuously during all of the weeks; the individuals checked either a single 4-week interval or multiple 4-week intervals in the questionnaire.

Crude results from analyzing the number of exposed intervals suggested that exposure to benzodiazepines and/or z-hypnotics in multiple 4-week intervals was associated with a higher risk of ADHD compared with exposure in a single 4-week interval. After weighting, the point estimates were attenuated (mental health sample: weighted HR, 1.32 [95% CI, 0.55-3.17]; full sample: weighted HR, 1.48 [95% CI, 0.63-3.45]) (Table 2). The 95% CIs included the null for both the crude and weighted analyses.

### Subgroup and Sensitivity Analyses

Baseline characteristics of the complete case full sample (n = 63 516) and the complete case mental health sample (n = 14 846) are presented in eTable 6 in Supplement 1. Exposure to benzodiazepine and/or z-hypnotics occurred in 497 pregnancies (0.8%) in the complete case full sample and 338 (2.3%) pregnancies in the complete case mental health sample. Results from the complete case analysis of ever exposure and timing of exposure during pregnancy did not differ substantially from the main results, whereas the estimates for number of exposed intervals were higher compared with the main results but were estimated with low precision (Table 3). Comparing the offspring of mothers who continued benzodiazepine and/or z-hypnotics use in pregnancy with the offspring of mothers who discontinued use before pregnancy revealed an increased risk of ADHD associated with benzodiazepine and/or z-hypnotic exposure during pregnancy, but the 95% CI included the null (weighted HR, 1.26; 95% CI, 0.56-2.85) (Figure 2).

**Table 3.** Associations of Prenatal Benzodiazepine and/or Z-Hypnotic Exposure With Child ADHD in the Complete Case Full and Mental Health Samples

	No. of offspring	Events No.	IR per 1000 person-years	HR (95% CI)	
				Crude	Weighted^a^
Complete case full sample (n = 63 516)					
Exposed anytime in pregnancy	497	14	2.5	1.08 (0.64-1.83)	0.89 (0.42-1.90)
Unexposed in pregnancy	63 019	1606	2.3	1 [Reference]	1 [Reference]
Timing					
Exposed, early pregnancy	308	10	2.9	1.21 (0.65-2.26)	0.51 (0.24-1.08)
Unexposed, early pregnancy	63 208	1610	2.3	1 [Reference]	1 [Reference]
Exposed, middle and/or late pregnancy	277	8	2.6	1.08 (0.54-2.18)	0.94 (0.43-2.10)
Unexposed, middle and/or late pregnancy	63 239	1612	2.3	1 [Reference]	1 [Reference]
No. of intervals^b^					
Exposed, single 4-wk interval	324	7	1.9	1 [Reference]	1 [Reference]
Exposed, multiple (≥2) 4-wk intervals	173	7	3.7	1.93 (0.68-5.44)	2.26 (0.73-7.00)
Complete case mental health sample (n = 14 846)					
Exposed anytime in pregnancy	338	12	3.2	0.95 (0.54-1.69)	0.83 (0.40-1.76)
Unexposed in pregnancy	14 508	521	3.3	1 [Reference]	1 [Reference]
Timing					
Exposed, early pregnancy	229	9	3.5	1.03 (0.53-2.01)	0.62 (0.27-1.43)
Unexposed, early pregnancy	14 617	524	3.3	1 [Reference]	1 [Reference]
Exposed, middle and/or late pregnancy	188	7	3.4	1.04 (0.48-2.21)	0.99 (0.39-2.50)
Unexposed, middle and/or late pregnancy	14 658	526	3.3	1 [Reference]	1 [Reference]
No. of intervals^b^					
Exposed, single 4-wk interval	196	5	2.3	1 [Reference]	1 [Reference]
Exposed, multiple (≥2) 4-wk intervals	142	7	4.5	2.01 (0.65-6.21)	2.41 (0.73-7.90)

Abbreviations: ADHD, attention-deficit/hyperactivity disorder; HR, hazard ratio, IR, incidence rate.

^a^ Inverse probability of treatment weights. Models for timing of exposure were additionally adjusted for depressive or anxiety symptoms (using the 5-item short version of the Hopkins Symptoms Checklist) because the weights could not balance that covariate.

^b^ The number of weeks did not imply that the pregnant individuals used the medication continuously during all of the weeks; the individuals checked either a single 4-week interval or multiple 4-week intervals in the questionnaires.

The complete case analysis, subgroup and sensitivity analyses, and probabilistic bias analysis showed that the association measures were generally robust (eResults, eTable 7, and eFigure 9 in Supplement 1). Results from estimating period-specific HRs differed slightly from the main analysis, especially the estimate for the first period, which was slightly higher (weighted HR, 0.85 [95% CI, 0.51-1.42] vs 1.31 [95% CI, 0.73-2.35]). Results from pooled logistic regression models did not change substantially from the main results (eResults and eFigure 10 in Supplement 1).

## Discussion

In this large, prospective cohort study of 82 201 pregnancies, of which 681 offspring were exposed to benzodiazepines and/or z-hypnotics, we found no increased risk of childhood ADHD associated with timing of benzodiazepine and/or z-hypnotic exposure during pregnancy or exposure during pregnancy regardless of timing. We observed a slightly increased risk of childhood ADHD after exposure in multiple 4-week intervals during pregnancy compared with exposure in a single 4-week interval. Hyperactivity and attention-deficit symptoms in children who were regularly exposed to benzodiazepines in utero were reported in an article from 1989,^51^ but a 2019 study did not support this finding.^23^ Results of the present study might indicate an increased risk of ADHD associated with increased number of exposed intervals, but the HRs were imprecise with 95% CIs including the null. Consequently, we cannot rule out the role of residual confounding and/or chance.

We believe the current study provides novel knowledge about the association of timing of prenatal exposure to benzodiazepines and z-hypnotics and number of exposed intervals with childhood ADHD. Two recent systematic reviews pointed out the need for further research on the risk of adverse neurodevelopmental outcomes in offspring after prenatal exposure to benzodiazepines and/or z-hypnotics.^17,18^ Eleven studies have evaluated behavioral disorders in children after prenatal exposure to benzodiazepines and/or z-hypnotics,^23,24,25,52,53,54,55,56,57,58,59^ with 7 of these papers published more than 10 years ago. Three studies examined overdoses associated with suicide attempts,^53,54,55^ which was out of scope for this current study.

Findings of this study were consistent with results showing that, in children aged 5 years, (1) middle and/or late pregnancy exposure was not associated with ADHD symptoms, as assessed with the Conners Parent Rating Scale (middle exposure: β = −0.04 [95% CI, −0.36 to 0.28]; late exposure: β = 0.08 [95% CI, −0.19, 0.35])^23^ and (2) exposure anytime during pregnancy was not associated with externalizing behavior (risk ratio, 1.13; 95% CI, 0.70-1.83).^25^ Both of these studies restricted the study population to pregnant individuals with an underlying anxiety or insomnia indication for treatment. In addition, the study results aligned with those suggesting that exposure during pregnancy was not associated with aggressive behavior or oppositional defiant disorder at 6 years of age (β = 0.23; 95% CI, −0.30 to 0.76).^24^ These previous studies used psychometric instruments to measure long-term neurodevelopmental outcomes, which may be subject to bias.^16^ Measuring these outcomes using psychiatric diagnoses are necessary to detect problems that reach the threshold of a clinical diagnosis.

Together, results from previous and current studies are clinically important because they provide a basis for clinicians and pregnant individuals when making well-balanced decisions regarding the appropriate treatment during pregnancy. These findings, however, must be replicated in studies with higher study power as well as in other countries and settings before inferences can be made from the findings.

### Strengths and Limitations

There are several strengths to this study, including the long follow-up time in the MoBa. We used methods to account for time-varying exposure, confounders, and missing data. A recent systematic review suggested the importance of assessing the timing and duration of benzodiazepine and z-hypnotic exposure during pregnancy, focusing on behavioral and emotional outcomes, and controlling for confounders.^17^ These key elements were all addressed in the present study. Moreover, we performed several sensitivity analyses to assess the robustness of the results, including adjusting for familial risk of ADHD using parental self-report of ADHD symptoms.^60^

This study also had some limitations that should be acknowledged. First, we relied on self-reported medication exposure during pregnancy, which was based on recall and willingness to disclose use. However, given that benzodiazepines and z-hypnotics are used episodically, self-reported data may be more valid than prescription data. Data from prescription fillings may not reflect the actual timing of use because benzodiazepines and z-hypnotics are not used consecutively from the dispensing date.^61^ In both scenarios, however, exposure misclassification could occur. A study that compared self-reported benzodiazepine-anxiolytics use in pregnancy with filled prescriptions for benzodiazepine-anxiolytics found a sensitivity of 45% and a specificity of 99.7%.^61^ Probabilistic bias analysis showed that potential exposure misclassification did not substantially alter the results. Moreover, symptoms of depression and anxiety were based on self-reporting and were not measured at baseline. Thus, residual confounding and additional measurement error cannot be fully ruled out.

Second, we had few exposed cases and low study power. Due to low sample size, we were not able to restrict the sample to births after 2007 to have complete follow-up of all offspring in both the NorPD and NPR. For the same reason, we did not estimate the impact of exposure timing separately for middle and late pregnancy nor the number of exposed intervals in more categories. Information regarding benzodiazepine or z-hypnotic dose was not available in the MoBa; consequently, we did not know whether a pregnant individual who reported use during a single 4-week interval used the medication only once or daily. The analysis of number of exposed intervals should be interpreted with caution. Third, the MoBa has a low response rate (41%), and the participants might not be representative of the general population.^62^

The current body of evidence is still insufficient for definitively establishing the neurodevelopmental safety for benzodiazepines and z-hypnotics.^17,18^ It is necessary to assess several domains of neurodevelopment and not only the risk of ADHD.^16,17^ Animal studies have indicated that learning outcomes also should be assessed in future studies.^17,63^

## Conclusions

This cohort study found no association between use of benzodiazepines and z-hypnotics during pregnancy and childhood ADHD in their offspring according to timing of exposure and number of exposed intervals. The study results provide reassurance for pregnant patients in need of benzodiazepines and z-hypnotics; however, these findings need to be interpreted with caution due to low study power in some of the analyses.

## Notes

Supplement 1.
eMethods.

eResults.
**eTable 1.** Overview of the Indications for Benzodiazepine and/or Z-Hypnotic Use in the MoBa Questionnaires Used to Create the Mental Health Sample **eTable 2.** Use of Benzodiazepines and Z-Hypnotics in the Different Time Windows **eTable 3**. Number of Intervals Exposed to Benzodiazepines and/or Z-Hypnotics During Pregnancy **eTable 4.** Overview of Co-Medication Exposure in the Different Time Windows **eTable 5.** Characteristics of the Generated Stabilized Weights **eTable 6.** Characteristics of Pregnancies by Exposure Status in the Complete Case Full and Mental Health Samples **eTable 7.** Specification of the Different Scenarios for the Probabilistic Bias Analyses and Results From Probabilistic Bias Analyses **eFigure 1.** Directed Acyclic Graph Showing Assumed Covariate Structure, Drawn as a Time-Fixed Model for Simplicity **eFigure 2.** Directed Acyclic Graph Showing Time-Varying Exposure and Confounding **eFigure 3.** Balance of Covariates Between Benzodiazepine and/or Z-Hypnotic Exposed and Unexposed Before and After IPTW **eFigure 4.** Balance of Covariates Benzodiazepine and/or Z-Hypnotic Exposed and Unexposed Before and After IPTW for Analyzing Number of Exposed Intervals **eFigure 5.** Kaplan-Meier Failure Curve Showing Cumulative Incidence of ADHD in Childhood **eFigure 6.** Crude Kaplan-Meier Failure Curves Showing Cumulative Incidence of ADHD in Childhood Among Benzodiazepine and/or Z-Hypnotic Exposed Compared to Unexposed **eFigure 7.** Weighted Kaplan-Meier Failure Curves Showing Cumulative Incidence of ADHD in Childhood Among Benzodiazepine and/or Z-Hypnotic Exposed Compared to Unexposed **eFigure 8.** Weighted Kaplan-Meier Failure Curves Showing Cumulative Incidence of ADHD in Childhood Among Benzodiazepine and/or Z-Hypnotic Exposed Compared to Unexposed in the Mental Health Sample **eFigure 9.** Results From Subgroup and Sensitivity Analyses **eFigure 10.** Survival Curves Standardized for Baseline Covariates and Baseline Values of Time-Varying Covariates

Supplement 2.
Data Sharing Statement
