# Supplementary material for: Association of Prenatal Exposure to Benzodiazepines and Z-Hypnotics With Risk of Attention-Deficit/Hyperactivity Disorder in Childhood
Source: JAMA Netw Open. 2022 Dec 15;5(12):e2246889. doi: 10.1001/jamanetworkopen.2022.46889 (PMC9856385; doi:10.1001/jamanetworkopen.2022.46889)
Supplement: Supplement 1. — eMethods. eResults. eTable 1. Overview of the Indications for Benzodiazepine and/or Z-Hypnotic Use in the MoBa Questionnaires Used to Create the Mental Health Sample eTable 2. Use of Benzodiazepines and Z-Hypnotics in the Different Time Windows eTable 3. Number of Intervals Exposed to Benzodiazepines and/or Z-Hypnotics During Pregnancy eTable 4. Overview of Co-Medication Exposure in the Different Time Windows eTable 5. Characteristics of the Generated Stabilized Weights eTable 6. Characteristics of Pregnancies by Exposure Status in the Complete Case Full and Mental Health Samples eTable 7. Specification of the Different Scenarios for the Probabilistic Bias Analyses and Results From Probabilistic Bias Analyses eFigure 1. Directed Acyclic Graph Showing Assumed Covariate Structure, Drawn as a Time-Fixed Model for Simplicity eFigure 2. Directed Acyclic Graph Showing Time-Varying Exposure and Confounding eFigure 3. Balance of Covariates Between Benzodiazepine and/or Z-Hypnotic Exposed and Unexposed Before and After IPTW eFigure 4. Balance of Covariates Benzodiazepine and/or Z-Hypnotic Exposed and Unexposed Before and After IPTW for Analyzing Number of Exposed Intervals eFigure 5. Kaplan-Meier Failure Curve Showing Cumulative Incidence of ADHD in Childhood eFigure 6. Crude Kaplan-Meier Failure Curves Showing Cumulative Incidence of ADHD in Childhood Among Benzodiazepine and/or Z-Hypnotic Exposed Compared to Unexposed eFigure 7. Weighted Kaplan-Meier Failure Curves Showing Cumulative Incidence of ADHD in Childhood Among Benzodiazepine and/or Z-Hypnotic Exposed Compared to Unexposed eFigure 8. Weighted Kaplan-Meier Failure Curves Showing Cumulative Incidence of ADHD in Childhood Among Benzodiazepine and/or Z-Hypnotic Exposed Compared to Unexposed in the Mental Health Sample eFigure 9. Results From Subgroup and Sensitivity Analyses eFigure 10. Survival Curves Standardized for Baseline Covariates and Baseline Values of Time-Varying Covariates [file jamanetwopen-e2246889-s001.pdf]

Supplemental Online Content

Sundbakk LM, Gran JM, Wood ME, Handal M, Skurtveit S, Nordeng H. Association of prenatal exposure to benzodiazepines and z-hypnotics with risk of attention-deficit/hyperactivity disorder in childhood. *JAMA Netw Open*. 2022;5(12):e2246889. doi:10.1001/jamanetworkopen.2022.46889

eMethods.

eResults.

eTable 1. Overview of the Indications for Benzodiazepine and/or Z-Hypnotic Use in the MoBa Questionnaires Used to Create the Mental Health Sample

eTable 2. Use of Benzodiazepines and Z-Hypnotics in the Different Time Windows

eTable 3. Number of Intervals Exposed to Benzodiazepines and/or Z-Hypnotics During Pregnancy

eTable 4. Overview of Co-Medication Exposure in the Different Time Windows

eTable 5. Characteristics of the Generated Stabilized Weights

eTable 6. Characteristics of Pregnancies by Exposure Status in the Complete Case Full and Mental Health Samples

eTable 7. Specification of the Different Scenarios for the Probabilistic Bias Analyses and Results From Probabilistic Bias Analyses

eFigure 1. Directed Acyclic Graph Showing Assumed Covariate Structure, Drawn as a Time-Fixed Model for Simplicity

eFigure 2. Directed Acyclic Graph Showing Time-Varying Exposure and Confounding

eFigure 3. Balance of Covariates Between Benzodiazepine and/or Z-Hypnotic Exposed and Unexposed Before and After IPTW

eFigure 4. Balance of Covariates Benzodiazepine and/or Z-Hypnotic Exposed and Unexposed Before and After IPTW for Analyzing Number of Exposed Intervals

eFigure 5. Kaplan-Meier Failure Curve Showing Cumulative Incidence of ADHD in Childhood

eFigure 6. Crude Kaplan-Meier Failure Curves Showing Cumulative Incidence of ADHD in Childhood Among Benzodiazepine and/or Z-Hypnotic Exposed Compared to Unexposed

eFigure 7. Weighted Kaplan-Meier Failure Curves Showing Cumulative Incidence of ADHD in Childhood Among Benzodiazepine and/or Z-Hypnotic Exposed Compared to Unexposed

© 2022 Sundbakk LM et al. *JAMA Network Open.*

eFigure 8. Weighted Kaplan-Meier Failure Curves Showing Cumulative Incidence of ADHD in Childhood Among Benzodiazepine and/or Z-Hypnotic Exposed Compared to Unexposed in the Mental Health Sample

eFigure 9. Results From Subgroup and Sensitivity Analyses

eFigure 10. Survival Curves Standardized for Baseline Covariates and Baseline Values of Time-Varying Covariates

This supplemental material has been provided by the authors to give readers additional information about their work.

© 2022 Sundbakk LM et al. *JAMA Network Open.*

eMethods

Covariates

The time-varying covariates were maternal symptoms of depression and anxiety during pregnancy and maternal co-medication use during pregnancy. Maternal symptoms of depression and anxiety during pregnancy were assessed with a validated short version of the Hopkins Symptom Checklist (SCL-5)[1] at gestational week 17. Mean summary scores were calculated and standardized to z-scores with mean of zero and a standard deviation of one. The mothers reported a number of concomitant medications in the MoBa questionnaires 1, 3 and 4: nonsteroidal anti-inflammatory drugs (NSAIDs; ATC code M01A), opioids (ATC code N02A), paracetamol (ATC code N02BE01), antidepressants (ATC code N06A), antipsychotics (ATC code N05A) and antiepileptics (ATC code N03A). Co-medication was categorized as occurring in early, middle and/or late pregnancy, during the 6 months before pregnancy, or at any time during gestation.

The baseline confounders included both maternal sociodemographic characteristics and maternal medical characteristics. Data on maternal and paternal age at delivery, parity, marital status, paternal education, folic acid intake before and during pregnancy, and year of birth were retrieved from the MBRN. The MoBa questionnaires provided data on body mass index (BMI) before conception, smoking, illicit drug use, alcohol intake, planned pregnancy, income, ongoing or completed education, adverse life events, obstetric comorbidity index, and lifetime history of major depression (LTH of MD). The mother's LTH of MD was reported according to five key depressive symptoms, which corresponded closely to the DSM-III criteria for lifetime major depression.[2] The obstetric comorbidity index is adapted from Bateman et al.[3] We used the following variables available in MBRN or MoBa: asthma, cardiac disease, kidney disease, congenital heart disease, drug abuse, placenta praevia, diabetes mellitus, chronic hypertension, gestational hypertension, previous cesarean section, lupus, age, severe preeclampsia. The variables were weighted as done by Bateman et al.

Additionally, the mothers reported previous and/or current illnesses and health problems on the MoBa Q1 questionnaires, which included sleeping problems. The NorPD provided information on maternal and paternal use of ADHD medication. We defined ADHD medication use similar as for the offspring. In addition, both mothers and fathers answered questions regarding symptoms of ADHD on the MoBa questionnaires, (Adult ADHD Self-Report Scale, ASRS). These variables were added to the model in sensitivity analyses (see Subgroup and sensitivity analyses**).**

Below follows a description of the instruments used in the analyses. An instrument documentation is available at https://www.fhi.no/en/studies/moba/for-forskere-artikler/questionnaires-from-moba/.

Severity of depression and anxiety symptoms: The Hopkins Symptoms Checklist-25 (SCL-25) is a psychometric scale designed to measure symptoms of anxiety and depression in population surveys[1]. SCL-5 is highly correlated to the SCL-25.[4,5] The SCL-5 composes of the following items: "Nervousness or shakiness inside", "Feeling fearful", "Feeling hopeless about the future", "Feeling blue" and "Worrying too much about things". Individuals could indicate whether they had been bothered by any of the listed symptoms during the last two weeks in a four category response, from "not bothered" to "very bothered", which are rated 1 to 4, respectively.

Adverse life events: Adverse life events from 6 months before pregnancy to third trimester were measured in Q3. Individuals were asked to indicate whether they had experienced different types of problems (e.g., at work or study place, financial, divorce) or events (e.g., a serious injury or accident, illness, abuse or loss of someone close) during the last 12 months, and how painful/difficult these problems and events were (not too bad; painful/difficult; very painful/difficult). Individuals were grouped as having experienced none, at least one not painful adverse life event, at least one painful or very painful event. The questions on the MoBa questionnaire were selected primarily because of their relevance to the population in general, partly due to their relevance to mothers with small children.

Adult ADHD Self-Report Scale (ASRS Screener): The scale is included in Q6 and for fathers (only version D of the father questionnaire). Adult ADHD Self-Report Scale[6] is a self-report screening scale of adult attention-deficit/hyperactivity disorder (ADHD). Four questions (1-4) capture symptoms of inattention and two questions (5 and 6) entail symptoms of hyperactivity - impulsivity. The response options are "never", "rarely", "sometimes", "often", and "very often". The self-administrating nature of the ASRS Screener and the small number of questions makes it a suitable instrument for screening in large population-based questionnaires and epidemiological studies.[6]

© 2022 Sundbakk LM et al. *JAMA Network Open.*

Statistical methods

The table below gives a description of the model specification for the inverse probability of treatment weights (IPTWs) for estimating the association between timing of benzodiazepine and/or z-hypnotic exposure and ADHD.

	**Description of IPTWs**
**Main analysis**	T1:<br>Numerator:<br>Probability of benzodiazepine and/or z-hypnotic use in early pregnancy given treatment of benzodiazepine and/or z-hypnotic before pregnancy<br>Denominator:<br>Probability of benzodiazepine and/or z-hypnotic use in early pregnancy given treatment of benzodiazepine and/or z-hypnotic before pregnancy<br>+<br>Time-varying covariates: co-medication use before pregnancy<br>+<br>Baseline covariates: Maternal age, marital status, parity, BMI, education, smoking, alcohol, income, parity, planned pregnancy, folic acid supplementation, illicit drugs, LTH of MD, adverse life events, sleeping problems, ADHD medication, obstetric comorbidity index.<br><br>T2:<br>Numerator:<br>Probability of benzodiazepine and/or z-hypnotic use in middle and/or late pregnancy given previous treatment<br>Denominator:<br>Probability of benzodiazepine and/or z-hypnotic use in middle and/or late pregnancy given<br>previous treatment<br>+<br>Time-varying covariates: Depressive/anxiety symptoms at gestational week 17 (SCL-5), co-medication use in early pregnancy.<br>+<br>Baseline covariates: Maternal age, marital status, parity, BMI, education, smoking, alcohol, income, parity, planned pregnancy, folic acid supplementation, illicit drugs, LTH of MD, adverse life events, sleeping problems, ADHD medication, obstetric comorbidity index.

Missing data

Under the assumption that data were missing at random, we imputed incomplete data via multiple imputation with chained equation[7-9] The imputation procedure included exposure and outcome variables, baseline hazard, and auxiliary variables (e.g., maternal sociodemographic variables and health related variables, co-medication). Imputed data were used in all analyses.[7-9]

Subgroup and sensitivity analyses

As mentioned in the manuscript, we restricted the full sample to mother-offspring dyads in which the mother used benzodiazepines and/or z-hypnotics during the 6 months before pregnancy and either continued or discontinued in pregnancy. The rationale behind this restriction, is that the mothers in this sample were likely to have similar mental health conditions, and we assumed that analyses of this sample would contribute to separating the effects of the underlying maternal conditions. This restriction created two mutually exclusive groups: (1) mothers who reported use of any benzodiazepine and/or z-hypnotic in the 6-month period before pregnancy, who continued treatment during pregnancy: (2) the discontinuers – mothers who reported use of any benzodiazepine and/or z-hypnotic in the 6-month period before pregnancy, who discontinued treatment during pregnancy.

© 2022 Sundbakk LM et al. *JAMA Network Open.*

The table below gives an overview of the different subgroup and sensitivity analyses we have performed.

Model	Description
1	Restricting the data to only one singleton pregnancy per individual, as some individuals participate with more than one pregnancy in the MoBa.
2	Restricting the data to births after 2003 (so that all offspring were followed up in the NorPD since birth).
3	Exclude offspring who died or emigrated during follow-up
4	Changing the outcome definition: restricting to the NPR records only.
5	Changing the outcome definition: restricting to the NPR records only, in addition to defining ADHD as at least two diagnoses of ADHD registered in the NPR.
6	Changing the outcome definition: defining ADHD as at least two diagnoses of ADHD registered in the NPR and/or one prescription of ADHD medication according to the NorPD.
7	Change the follow-up time for the offspring who did not experience the event of interest: 2016 – birth year.
8	Change the follow-up time for the offspring who did not experience the event of interest: 2017 – birth year.
9	Changing the exposure definition: BZD-anxiolytics (N05BA).
10	Changing the exposure definition: z-hypnotics (N05CF).
11	Changing the exposure definition: BZD-anxiolytics (N05BA), BZD-hypnotics (N05CD) and/or z-hypnotics (N05CF).
12	Adding information to the main model: paternal ASRS, ADHD medication, SCL, LTH of MD, education and age, in addition to maternal ASRS.

Abbreviations: ADHD, attention deficit/hyperactivity disorder; ASRS, Adult ADHD Self-Report Scale; BZD, benzodiazepine; LTH of MD, Life Time History of Major Depression; MoBa, The Norwegian Mother, Father and Child cohort study; NorPD, Norwegian Prescription Database; NPR, Norwegian Patient Registry.

Below follows a description of the rationale for each subgroup and sensitivity analysis.

First, we restricted the data samples into a sample with only one singleton pregnancy per individual (model 1). The MoBa started following offspring from 1999, and the NorPD contains information about prescribed drugs since 2004. This means that offspring born before 2004 entered the study at age four years at the latest. To address the potential bias due to this, we replicated the analyses in a sub-sample of mother-offspring dyads with birth year 2004 or after (model 2). The current study did not have information about dates of potential offspring death or emigration. To check for potential bias of misclassified time at risk, we excluded offspring who died or emigrated during follow-up (model 3).

In order to check for bias due to misclassification of the outcome, we performed the following sensitivity analyses: Some offspring with a specialist diagnosis of ADHD do not use medication for ADHD. Thus, we performed an analysis changing the outcome definition into only a diagnosis according to the NPR (model 4), rather than the definition used in the main model: an ADHD diagnosis and/or a filled prescription for ADHD medication. In addition, in model 5, we used a stricter definition of ADHD, whereby offspring were classified as having the ADHD outcome if they had at least two diagnoses of ADHD registered in the NPR. In model 6, we

© 2022 Sundbakk LM et al. *JAMA Network Open.*

classified offpsring as having the ADHD outcome if they had at least two diagnoses of ADHD registered in the NPR and/or at least one prescription of ADHD medication according to the NorPD.

Information about the date of birth for the offspring who did not experience the outcome of interest in this study, was not available, only the year of birth. In order to check for potential bias due to defining the time at risk for the offspring who did not experience the event of interest in this study, we re-defined the follow-up time as subtracting the birth year from 2016 (model 7) and 2017 (model 8).

Additionally, we investigated the potential impact of exposure misclassification. In three different models we changed the exposure definition to: benzodiazepine-anxiolytics (model 9), z-hypnotics (model 10) and benzodiazepine-anxiolytics, benzodiazepine-hypnotics and/or z-hypnotics (model 11). Lastly, we conducted models that took into account additional maternal and paternal factors (model 12).

We used probabilistic bias analysis in order to handle potential exposure misclassification.[10,11] Exposure misclassification was considered non-differential. A previous study comparing self-reported data on benzodiazepine use in the MoBa to data for dispensed drugs in the NorPD, found that sensitivity was 0.45 and specificity 0.997 for benzodiazepine-anxiolytics (ATC code N05BA) use in pregnancy.[12] Expanding the time window for dispensed drugs to include 30 and 60 days before pregnancy led to higher sensitivity, 0.51 and 0.53, respectively, and lower specificity, 0.995 and 0.994, respectively. For benzodiazepine-hypnotics use in pregnancy (ATC code N05CD) the sensitivity was 0.28, and specificity 100. Expanding the time window for dispensed drugs to include 30 and 60 days before pregnancy led to higher sensitivity, 0.33 for both time windows, and unchanged specificity. We specified trapezoidal distributions of the bias parameters. eTable 7 shows the different scenarios of sensitivity and specificity value. For specificity, we did not use lower minimums, as that resulted in negative adjusted counts.

We repeated the main analysis after excluding individuals from regions of the propensity score distribution with no overlap, to avoid violations of the positivity assumption.[13,14]

Period specific cox regression models

In eFigure 7 and 8, we see that the weighted Kaplan-Meier failure curves, showing cumulative incidence of ADHD in childhood among benzodiazepine and/or z-hypnotic exposed compared to unexposed, cross, which may indicate that the proportional hazards assumption is not met. We split the follow-up time at 9.53 years, which is the time point where the curves cross. We created stabilized IPTWs for each time period applied to cox regression models, in order to estimate period specific weighted hazard ratios (HRs).

Pooled logistic regression models

In addition to estimating period specific weighted HRs, we applied a pooled logistic regression model to the complete case full sample, which allowed us to estimate the baseline hazard in a more flexible manner.[15] In order to estimate conditional causal effects, we used pooled logistic regression models to calculate crude and covariate-adjusted HRs. We included a variable for time of follow up, in addition to time of follow-up as a quadratic polynomial. In order to estimate marginal causal effects, we standardized over baseline covariates and baseline values of time-varying covariates. We included the time of follow-up as a quadratic polynomial, as well as interaction terms between exposure and the time variables. We calculated standardized survival curves (one for survival if everyone was exposed, one for survival if everyone was unexposed) and the marginal cumulative incidence ratio after end of follow-up.

© 2022 Sundbakk LM et al. *JAMA Network Open.*

References

1. Strand BH, Dalgard OS, Tambs K, Rognerud M. Measuring the mental health status of the Norwegian population: A comparison of the instruments SCL-25, SCL-10, SCL-5 and MHI-5 (SF-36). *Nord J Psychiatry.* 2003;57(2):113-118.
2. Kendler KS, Neale MC, Kessler RC, Heath AC, Eaves LJ. The lifetime history of major depression in women: Reliability of diagnosis and heritability. *Arch Gen Psychiatry.* 1993;50(11):863-870.
3. Bateman BT, Mhyre JM, Hernandez-Diaz S, et al. Development of a comorbidity index for use in obstetric patients. *Obstet Gynecol.* 2013;122(5):957-965.
4. Tambs K, Moum T. How well can a few questionnaire items indicate anxiety and depression? *Acta Psychiatr Scand.* 1993;87(5):364-367.
5. Fink P, Ørnbøl E, Huyse FJ, et al. A brief diagnostic screening instrument for mental disturbances in general medical wards. *J Psychosom Res.* 2004;57(1):17-24.
6. Kessler RC, Adler L, Ames M, et al. The World Health Organization adult ADHD self-report scale (ASRS): a short screening scale for use in the general population. In. *Psychol. Med.*2005:245-256.
7. Rubin DB. *Multiple imputation for nonresponse in surveys.* New York: Wiley; 1987.
8. Sterne JA, White IR, Carlin JB, et al. Multiple imputation for missing data in epidemiological and clinical research: potential and pitfalls. *BMJ.* 2009;338:b2393.
9. Moodie EE, Delaney JA, Lefebvre G, Platt RW. Missing confounding data in marginal structural models: a comparison of inverse probability weighting and multiple imputation. *The international journal of biostatistics.* 2008;4(1):Article 13.
10. Lash TL, Fox MP, Fink AK. *Applying Quantitative Bias Analysis to Epidemiologic Data.* New York, NY: Springer New York; 2009.
11. Lash TL, Fox MP, MacLehose RF, Maldonado G, McCandless LC, Greenland S. Good practices for quantitative bias analysis. *Int J Epidemiol.* 2014;43(6):1969-1985.
12. Skurtveit S, Selmer R, Odsbu I, Handal M. Self-reported data on medicine use in the Norwegian Mother and Child Cohort Study compared to data from the Norwegian Prescription Database. *Nor Epidemiol.* 2014;24(1-2):209-216.
13. Rosenbaum PR, Rubin DB. The central role of the propensity score in observational studies for causal effects. *Biometrika.* 1983;70(1):41-55.
14. Austin PC. An introduction to propensity score methods for reducing the effects of confounding in observational studies. *Multivariate Behav Res.* 2011;46(3):399-424.
15. Hernán MA. The hazards of hazard ratios. *Epidemiology (Cambridge, Mass).* 2010;21(1):13-15.

© 2022 Sundbakk LM et al. *JAMA Network Open.*

eResults

Missing data

The tables below show the comparison of the characteristics with incomplete data before and after multiple imputation (MI), for categorical and continuous variables respectively.

Categorical variables:

**Characteristics**	**Before MI**		**After MI[a]**
**Maternal characteristics**	**No.**	**% of N=82 201**	**% of N=20*82 201**
College or university educational level	53 710	65.6	65.6
Smoking during pregnancy	6188	7.6	7.7
Alcohol intake during pregnancy	9606	12.7	12.7
Gross yearly income			
Average	62 804	78.9	78.9
Low	7866	9.9	10.0
High	8912	11.2	11.1
Planned pregnancy	67 306	82.0	82.0
LTH of MD	4816	6.0	6.0
**Paternal characteristics**			
Age			
< 25 years	3582	4.4	4.4
25-39 years	70 324	85.7	85.7
40-49 years	7587	9.3	9.3
> 49 years	518	0.6	0.6
College or university educational level	40 465	49.7	49.5
LTH of MD	6206	10.0	10.2

Abbreviations: LTH of MD, Life Time History of Major Depression; MI, multiple imputation.
[a]Mean proportion the 20 imputed datasets.

Continuous variables:

**Characteristics**	**Before MI**	**After MI[a]**
**Maternal characteristics**	**mean ± SD**	**mean ± SD**
Pre-pregnancy BMI	24.0 ± 4.2	24.0 ± 4.2
Depressive or anxiety symptoms during pregnancy, at week 17	0 ± 1	0 ± 1
ADHD symptom level (ASRS)	1.1 ± 0.6	1.1 ± 0.6
Obstetric comorbidity index	0.5 ± 1.0	0.5 ± 1.0
**Paternal characteristics**		
Depressive or anxiety symptoms during pregnancy	0 ± 1	0 ± 1
ADHD symptom level (ASRS)	1.4 ± 0.5	1.4 ± 0.5

Abbreviations: ADHD, attention-deficit/hyperactivity disorder; ASRS, Adult ADHD Self-Report Scale; BMI, body mass index; MI, multiple imputation; SCL-5, the Hopkins Symptoms Checklist-5; SD, standard deviation.
[a]Mean of the 20 means from each of the 20 imputed dataset.

© 2022 Sundbakk LM et al. *JAMA Network Open.*

Subgroup and sensitivity analyses

Restricting to only one singleton pregnancy per individual (model 1: full sample n=69 582, mental health sample n=17 070, excluded 15.4% and 12.8%, respectively) did not alter the findings. By restricting to mother-offspring dyads with birth year 2004 or after (model 2: full sample n=60 625, mental health sample n=14 857, excluded 26.2% and 24.1%, respectively), the estimates were attenuated with the exception of the association between early pregnancy exposure to benzodiazepine and/or z-hypnotics and risk of ADHD in childhood in the mental health sample (weighted HR 1.46, 95% CI 0.75-2.85). When we excluded offspring who died or emigrated during follow-up (model 3: full sample n=81 489, mental health sample n=19 376, excluded 0.9% and 1.1%, respectively), we observed slightly higher point estimates for early and middle/late exposure in the mental health sample, compared to the main analyses. Results from sensitivity analyses to check for potential bias due to misclassification of the outcome (model 4-8) did not differ substantially from the findings in the main analysis. Nor did the results from sensitivity analyses to check for potential impact of exposure misclassification (model 9-11). See eTable 2 for numbers of exposed in the different exposure groups. Taking into account additional maternal and paternal factors (model 12) did not change the results. Results from all models are shown in eFigure 9.

Results from the probabilistic bias analysis showed that based on the three different scenarios of values for the sensitivity and specificity, potential exposure misclassification could have biased our results by 0.9-3.6%.

When analyzing only individuals in the overlapping regions of the propensity score distribution, we found effect estimates almost identical to the results in the main analyses.

Period specific cox regression models

The table below summarizes the results from the period specific cox regression models.

	No.	No. exposed	Crude HR (95% CI)	Weighted HR (95% CI)
**Age < 9.53 years**	25 674	221	2.16 (1.42-3.28)	1.31 (0.73-2.35)
**Age ≥ 9.53 years**	56 527	460	0.96 (0.43-2.15)	0.58 (0.20-1.72)

Abbreviations: CI, confidence interval; HR, hazard ratio.

Pooled logistic regression models

The results from estimating conditional causal effects from pooled logistic regression models (crude HR 1.10, 95% CI 0.65-1.86; covariate-adjusted conditional HR 0.71, 95% CI 0.41-1.22), did not differ substantially from the complete case analysis from cox regression models (Table 3 in the manuscript). The marginal cumulative incidence ratio after end of 17 years of follow-up was 0.71.

The table below shows results from covariate-adjusted pooled regression models standardizing over the baseline covariate distribution. eFigure 10 shows survival curves standardized for baseline covariates and baseline values of time-varying covariates. We see that the curves cross at around 9 years.

Years of follow-up	Average standardized survival for the unexposed	Average standardized survival for the exposed	Marginal risk difference	Standardized hazard ratio (HR)
**0 (Baseline)**	1.000	1.000	0.000	-
**1**	1.000	1.000	0.000	82.932
**2**	1.000	1.000	0.000	39.323
**3**	1.000	1.000	0.000	18.540
**4**	1.000	0.999	0.001	9.211
**5**	1.000	0.998	0.001	4.946
**6**	0.999	0.997	0.002	2.900
**7**	0.997	0.995	0.002	1.861
**8**	0.994	0.993	0.002	1.307
**9**	0.990	0.990	0.000	1.000
**10**	0.985	0.987	-0.003	0.828
**11**	0.979	0.985	-0.005	0.736

© 2022 Sundbakk LM et al. *JAMA Network Open.*

**12**	0.975	0.983	-0.008	0.691
**13**	0.972	0.981	-0.009	0.676
**14**	0.970	0.979	-0.010	0.678
**15**	0.969	0.978	-0.010	0.687
**16**	0.968	0.978	-0.009	0.696
**17**	0.968	0.978	-0.009	0.703

© 2022 Sundbakk LM et al. *JAMA Network Open.*

eTable 1. Overview of the indications for benzodiazepine and/or z-hypnotic use in the MoBa questionnaires used to create the mental health sample.

Indication	Q1	Q3	Q4
Depression	X	X	
Anxiety	X		
Other psychological problems	X (only version A)	X	
Sleeping problems	X		
Mental health problems			X

Q indicates questionnaire.

© 2022 Sundbakk LM et al. *JAMA Network Open.*

eTable 2. Use of benzodiazepines and z-hypnotics in the different time windows, No. (% of N).

	Exposure during the 6 months before pregnancy	Early pregnancy exposure	Middle and/or late pregnancy exposure	Exposure anytime during pregnancy
**Full sample (N=82 201)**				
Benzodiazepines				
Benzodiazepine-anxiolytics[a]	364 (0.44)	222 (0.27)	207 (0.25)	332 (0.40)
Benzodiazepine-hypnotics[b]	49 (0.06)	29 (0.04)	32 (0.04)	48 (0.06)
Benzodiazepine-antiepileptics[c]	17 (0.02)	17 (0.02)	9 (0.01)	19 (0.02)
Z-hypnotics[d]	271 (0.33)	200 (0.24)	147 (0.18)	255 (0.31)
Any benzodiazepine and/or z-hypnotic	637 (0.77)	435 (0.53)	374 (0.45)	681 (0.83)
**Mental health sample (N=19 585)**				
Benzodiazepines				
Benzodiazepine-anxiolytics[a]	265 (1.35)	163 (0.83)	144 (0.74)	233 (1.19)
Benzodiazepine-hypnotics[b]	31 (0.16)	19 (0.10)	18 (0.09)	28 (0.14)
Benzodiazepine-antiepileptics[c]	11 (0.06)	12 (0.06)	8 (0.04)	14 (0.07)
Z-hypnotics[d]	195 (1.00)	159 (0.81)	110 (0.56)	194 (0.99)
Any benzodiazepine and/or z-hypnotic	446 (2.3)	321 (1.6)	261(1.3)	468 (2.4)

[a] N05BA (diazepam, oxazepam, alprazolam)
[b] N05CD (nitrazepam, flunitrazepam, midazolam)
[c] N03AE01 (clonazepam)
[d] N05CF (zopiclone, zolpidem).

© 2022 Sundbakk LM et al. *JAMA Network Open.*

eTable 3. Number of intervals exposed to benzodiazepines and/or z-hypnotics during pregnancy, No. (% of N).

	Full sample (N=82 201)	Mental health sample (N= 19 585)
**Use in a single 4-week intervals during pregnancy**	436 (0.5)	268 (1.4)
**Use in multiple (≥2) 4-week intervals during pregnancy**	245 (0.3)	200 (1.0)

© 2022 Sundbakk LM et al. *JAMA Network Open.*

eTable 4. Overview of co-medication exposure in the different time windows, No. (% of N).

	**Exposed to benzodiazepines and/or z-hypnotics during pregnancy (N=681)**	**Not exposed to benzodiazepines or z-hypnotics during pregnancy (N=81 520)**
**Co-medication exposure (ATC code)**		
**NSAIDs (M01A)**		
Exposure during the 6 months before pregnancy	96 (14.1)	7604 (9.3)
Early pregnancy exposure	74 (10.9)	4064 (5.0)
**Opioids (N02A)**		
Exposure during the 6 months before pregnancy	45 (6.6)	1138 (1.4)
Early pregnancy exposure	63 (9.3)	996 (1.2)
**Antidepressants (N06A)**		
Exposure during the 6 months before pregnancy	93 (13.6)	1014 (1.2)
Early pregnancy exposure	92 (13.5)	682 (0.8)
**Antiepileptics[a] (N03A)**		
Exposure during the 6 months before pregnancy	11 (1.6)	227 (0.3)
Early pregnancy exposure	10 (1.5)	232 (0.3)
**Antipsychotics (N05A)**		
Exposure during the 6 months before pregnancy	19 (2.8)	129 (0.2)
Early pregnancy exposure	32 (4.7)	547 (0.7)
**Paracetamol (N02BE01)**		
Exposure during the 6 months before pregnancy	217 (31.9)	20 674 (25.4)
Early pregnancy exposure	369 (54.2)	30 037 (36.8)

Abbreviations: ATC code, Anatomical Therapeutic Chemical code; NSAIDs, nonsteroidal anti-inflammatory drug.
[a]Antiepileptics did not include clonazepam (N03AE01).

© 2022 Sundbakk LM et al. *JAMA Network Open.*

eTable 5. Characteristics of the generated stabilized weights[a].

	Full sample	Mental health sample
**Ever exposure**		
Mean (SD)	1.00 (0.10)	1.00 (0.14)
Min – max	0.01 – 5.87	0.03 – 4.96
**Timing of exposure**		
Mean (SD)	1.00 (0.11)	1.00 (0.14)
Min – max	0.01 – 10.47	0.05 – 9.93
**Number of exposed intervals**		
Mean (SD)	1.00 (0.39)	1.00 (0.38)
Min – max	0.38 – 3.95	0.47 – 3.25

Abbreviations: Max, maximum; Min, minimum; SD, standard deviation.
[a]Mean, SD, min and max from each of the 20 imputed datasets, then mean of the 20 values.

© 2022 Sundbakk LM et al. *JAMA Network Open.*

eTable 6. Characteristics of pregnancies by exposure status, in the complete case full and mental health samples.

Characteristic	Individuals by benzodiazepine and/or z-hypnotic exposure, No. (%)			
	Complete case full sample (N=63 516)		Complete case mental health sample (N=14 846)	
	Exposed	Unexposed	Exposed	Unexposed
**Maternal characteristics**				
No. of participants	497 (0.8)	63 019 (99.2)	338 (2.3)	14 508 (97.7)
Age in years, mean (SD)	31.2 (4.7)	30.3 (4.4)	31.4 (4.6)	30.3 (4.7)
Married or cohabiting	461(92.8)	61 155 (97.0)	305 (90.2)	13 867 (95.6)
Primiparous	233 (46.9)	29 252 (46.4)	181 (53.6)	7096 (48.9)
Pre-pregnancy BMI, mean (SD)	23.9 (4.2)	24.0 (4.2)	24.2 (4.5)	24.1 (4.4)
College or university educational level[a]	333 (67.0)	42 896 (68.1)	216 (63.9)	9193 (63.4)
Smoking during pregnancy	72 (14.5)	4222 (6.7)	53 (15.7)	1258 (8.7)
Alcohol intake during pregnancy	124 (24.9)	7559 (12.0)	89 (26.3)	1899 (13.1)
Gross yearly income[b]				
Average	382 (76.9)	49 422 (78.4)	262 (77.6)	11 049 (76.2)
Low	53 (10.6)	6211 (9.9)	38 (11.2)	1828 (12.6)
High	62 (12.5)	7386 (11.7)	38 (11.2)	1631 (11.2)
Planned pregnancy	374 (75.3)	52 273 (82.9)	251 (74.3)	11 529 (79.5)
Folic acid supplementation	297 (59.8)	39 214 (62.2)	205 (60.7)	9074 (62.5)
Illicit drug use[d]	16 (3.2)	307 (0.5)	11 (3.3)	136 (0.9)
Sleeping problems	215 (43.3)	9385 (14.9)	215 (63.6)	9835 (67.8)
Lifetime history of major depression, n (% of N)	97 (19.5)	3608 (5.7)	88 (26.0)	2045 (14.1)
Depressive or anxiety symptoms during pregnancy[e] at week 17, mean (SD) z-score	1.0 (1.7)	0 (1.0)	0.6 (1.4)	0 (1.0)

© 2022 Sundbakk LM et al. *JAMA Network Open.*

Characteristic	Individuals by benzodiazepine and/or z-hypnotic exposure, No. (%)			
	Complete case full sample (N=63 516)		Complete case mental health sample (N=14 846)	
	Exposed	Unexposed	Exposed	Unexposed
**Maternal characteristics**				
Adverse life event				
No	121 (24.3)	25 633 (40.7)	57 (16.9)	4047 (27.9)
At least 1: not painful	97 (19.5)	15 596 (24.7)	68 (20.1)	3619 (24.9)
At least 1: painful/very painful	279 (56.1)	21 790 (34.6)	213 (63.0)	6842 (47.2)
Co-medication use anytime during pregnancy				
NSAIDs	80 (16.1)	3974 (6.3)	57 (16.9)	1177 (8.1)
Opioids	68 (13.7)	1198 (1.9)	43 (12.7)	412 (2.8)
Paracetamol	333 (67.0)	28 510 (45.2)	225 (66.6)	7369 (50.8)
Antidepressants	85 (17.1)	577 (0.9)	84 (24.9)	543 (3.7)
Antipsychotics	34 (6.8)	468 (0.7)	30 (8.9)	182 (1.3)
Antiepileptics[f]	7 (1.4)	200 (0.3)	5 (1.5)	63 (0.4)
Obstetric comorbidity index[g], mean (SD)	0.8 (1.3)	0.5 (1.0)	0.9 (1.3)	0.6 (1.0)
ADHD prescriptions[h]	16 (3.2)	524 (0.8)	12 (3.6)	257 (1.8)

Abbreviations: ADHD, attention-deficit/hyperactivity disorder; BMI, body mass index (calculated as weight in kilograms divided by height in meters squared); NSAIDs, nonsteroidal anti-inflammatory drugs; SD, standard deviation.
[a]Included completed or ongoing education.
[b]Average income indicates approximately between US$14 214 to $47 759; low income indicates $14 214 or less; high income indicates $ 46 801 or higher.
[c]Folic acid supplementation before pregnancy or during pregnancy.
[d]Illicit drug use during pregnancy or the last month before pregnancy.
[e]Presence of depressive or anxiety symptoms measured with the 5-item short version of the Hopkins Symptoms Checklist (SCL-5).
[f]Antiepileptics did not include clonazepam (N03AE01).
[g]The obstetric comorbidity index was adapted from Bateman et al.[3] The following variables available in the Medical Birth Registry of Norway or Norwegian Mother, Father and Child Cohort Study were used: asthma, cardiac disease, kidney disease, congenital heart disease, drug abuse, placenta praevia, diabetes, chronic hypertension, gestational hypertension, previous cesarean delivery, lupus, age and severe preeclampsia. The variables were weighted consistent with Bateman et al.
[h]Indicates filled prescription for ADHD medication.

© 2022 Sundbakk LM et al. *JAMA Network Open.*

eTable 7. Specification of the different scenarios for the probabilistic bias analyses, and results from probabilistic bias analyses[a].

	Sensitivity				Specificity				Adjusted RR (95% CI)	Magnitude of bias (%)
	Min	Mode 1	Mode 2	Max	Min	Mode 1	Mode 2	Max		
**Scenario 1**	0.30	0.40	0.50	0.85	0.994	0.995	0.996	0.999	1.13 (0.67-1.90)	1.8%
**Scenario 2**	0.11	0.28	0.54	0.70	0.991	0.993	0.994	0.995	1.15 (0.68-1.96)	3.6%
**Scenario 3**	0.43	0.48	0.58	0.95	0.996	0.997	0.998	1.00	1.12 (0.66-1.91)	0.9%

Abbreviations: CI, confidence interval; Max, maximum; Min, minimum; RR, risk ratio.
[a]Conventional RR (95% CI) = 1.11 (0.65-1.88).

© 2022 Sundbakk LM et al. *JAMA Network Open.*

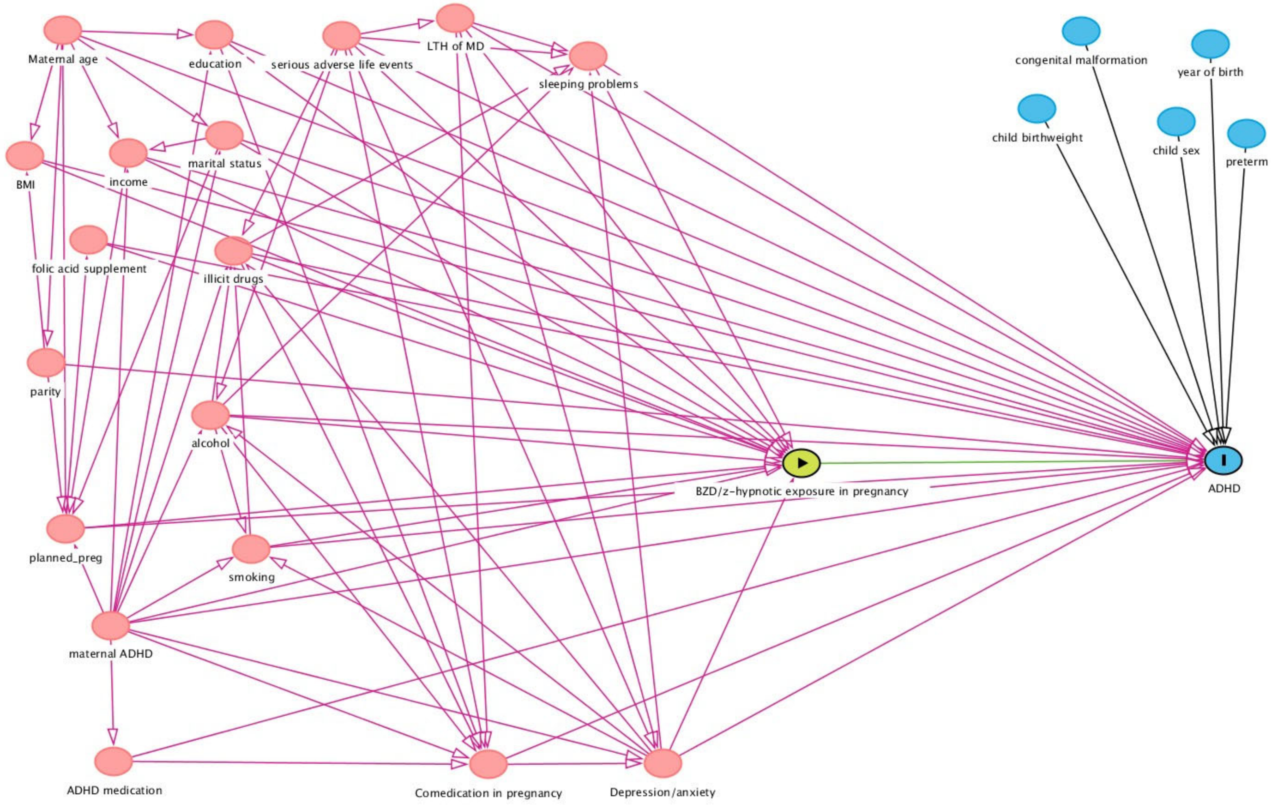

eFigure 1. Directed acyclic graph showing assumed covariate structure, drawn as a time-fixed model for simplicity.

Abbreviations: ADHD, Attention-Deficit/Hyperactivity Disorder; BMI, body mass index; BZD, benzodiazepine; LTH of MD, Life Time History of Major Depression.

© 2022 Sundbakk LM et al. *JAMA Network Open.*

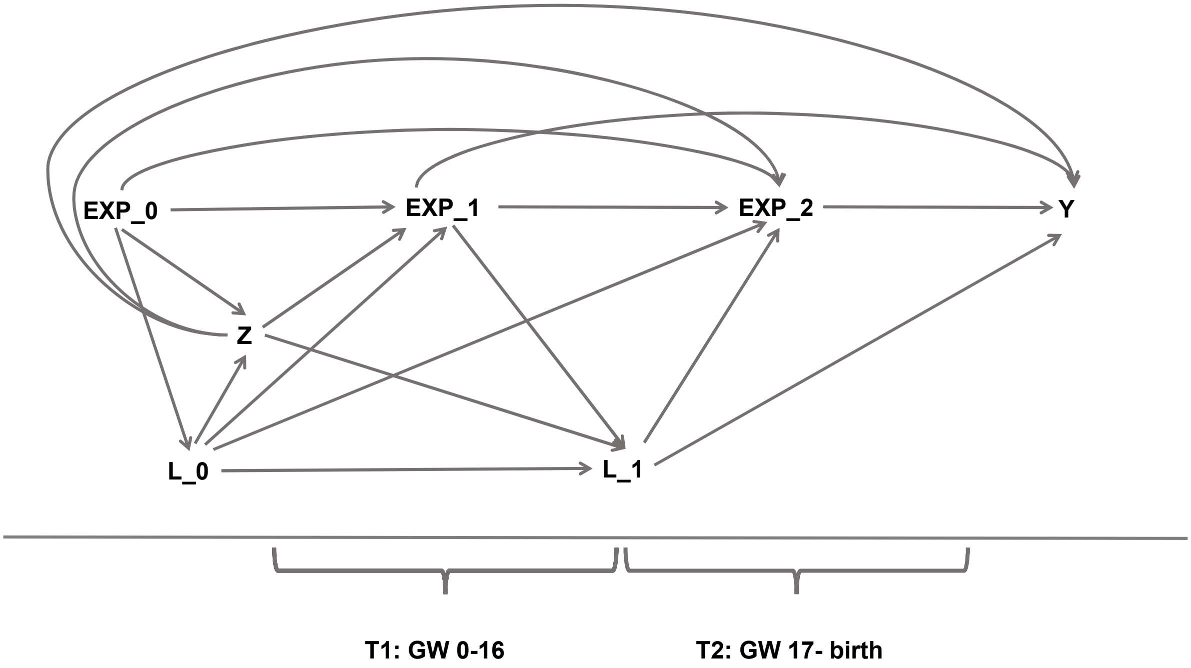

eFigure 2. Directed acyclic graph showing time-varying exposure and confounding. Z is a vector of baseline covariates; EXP_0 represent exposure to benzodiazepines and/or z-hypnotics during the 6 months before pregnancy, and EXP_1 and EXP_2 represent exposure to benzodiazeines and/or z-hypnotics in GW 0-16 (early) and GW 17-28 and/or 29-birth (mid and/or late)); L_0 is a vector of pre-pregnancy time-varying confounders including comedications; L_1 is a vector of time-varying confounders including comedication and depressive/anxiety symptoms measured at GW 17. Y is the outcome, ADHD in childhood. Abbreviations: ADHD, Attention-Deficit/Hyperactivity Disorder; GW, gestational week; PP, pre-pregnancy.

© 2022 Sundbakk LM et al. *JAMA Network Open.*

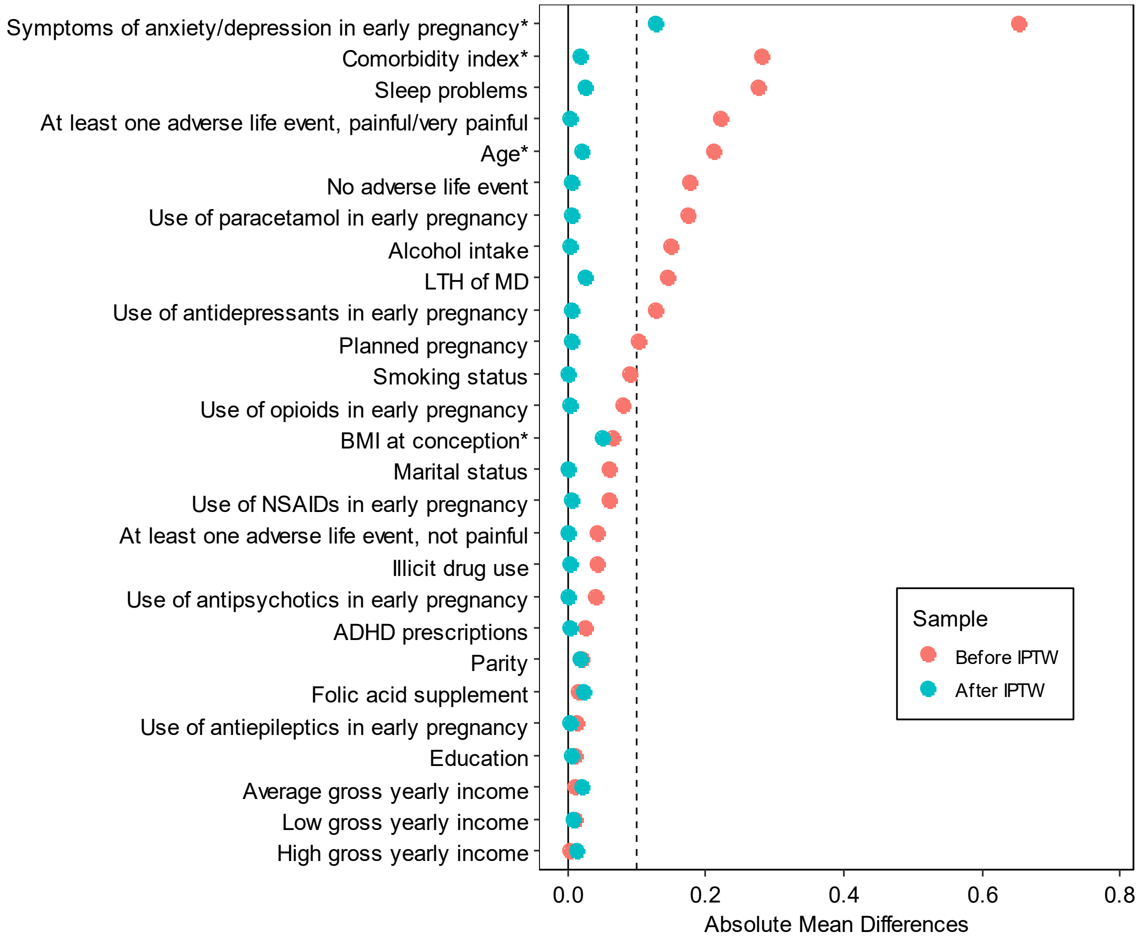

eFigure 3. Balance of covariates between benzodiazepine and/or z-hypnotic exposed and unexposed before and after IPTW. * indicates variables for which the displayed value is the standardized mean difference. Un-starred variables have raw mean differences displayed; starred variables have

© 2022 Sundbakk LM et al. *JAMA Network Open.*

standardized mean differences displayed. Abbreviations: ADHD, Attention-Deficit/Hyperactivity Disorder; BMI, body mass index; IPTW, inverse probability of treatment weighting; LTH of MD, Life Time History of Major Depression; NSAIDs, nonsteroidal anti-inflammatory drugs.

© 2022 Sundbakk LM et al. *JAMA Network Open.*

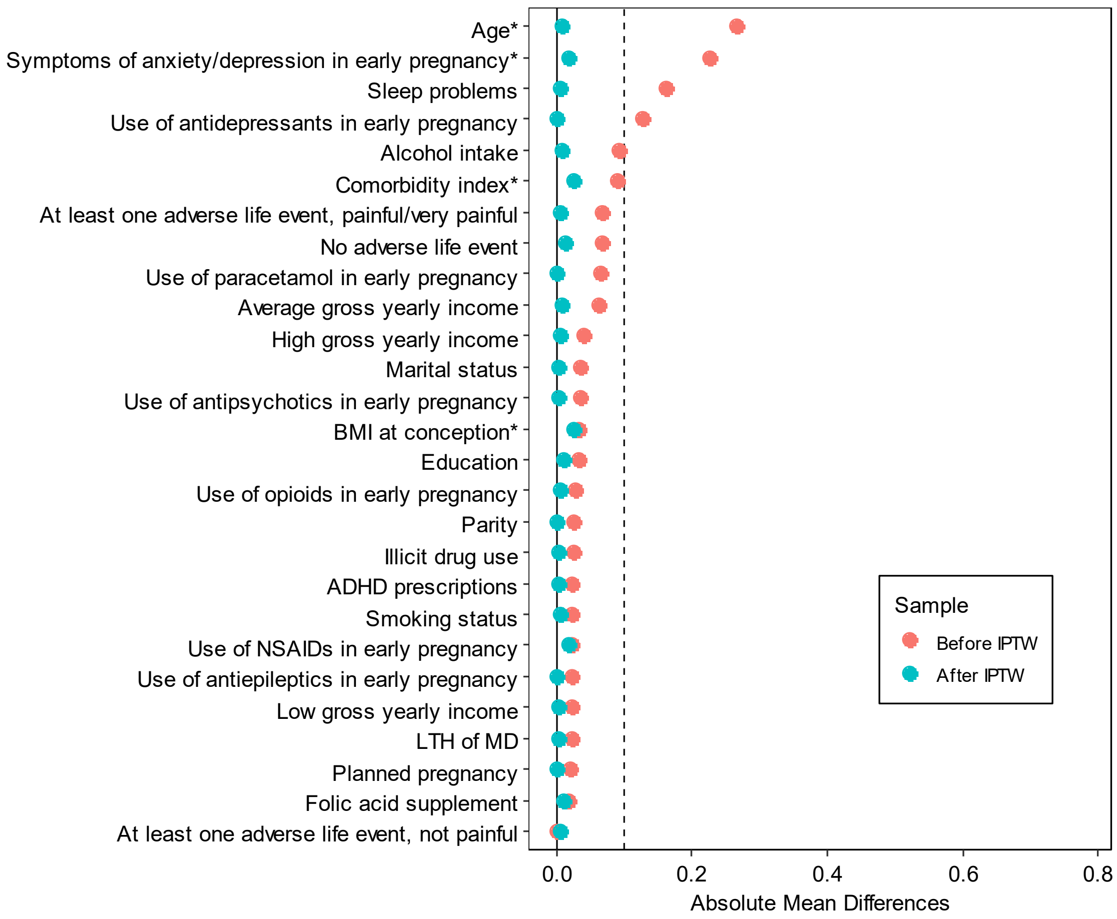

eFigure 4. Balance of covariates between benzodiazepine and/or z-hypnotic exposed and unexposed before and after IPTW, for analyzing number of exposed intervals. * indicates variables for which the displayed value is the standardized mean difference. Un-starred variables have raw mean differences displayed; starred variables have standardized mean differences displayed. Abbreviations: ADHD, Attention-Deficit/Hyperactivity Disorder; BMI,

© 2022 Sundbakk LM et al. *JAMA Network Open.*

body mass index; IPTW, inverse probability of treatment weighting; LTH of MD, Life Time History of Major Depression; NSAIDs, nonsteroidal anti-inflammatory drugs.

© 2022 Sundbakk LM et al. *JAMA Network Open.*

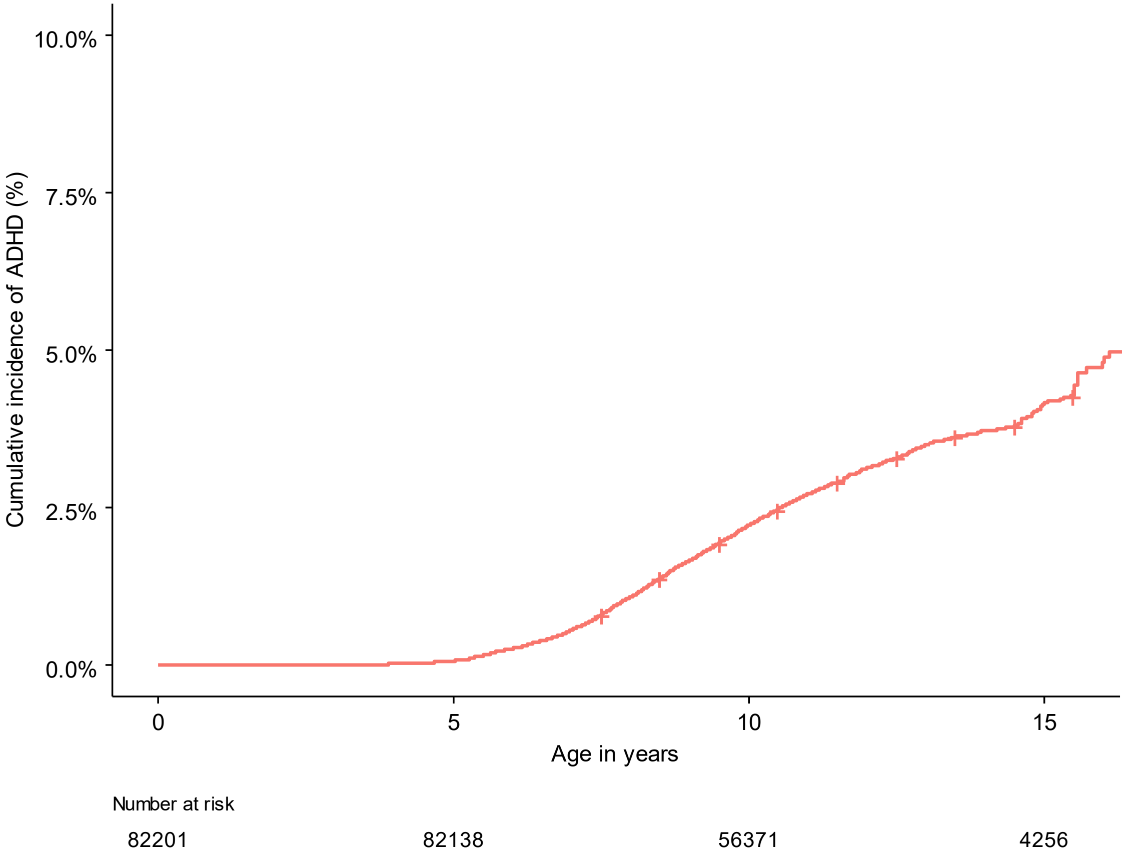

eFigure 5. Kaplan-Meier failure curve showing cumulative incidence of ADHD in childhood.

Abbreviation: ADHD, Attention-Deficit/Hyperactivity Disorder.

© 2022 Sundbakk LM et al. *JAMA Network Open.*

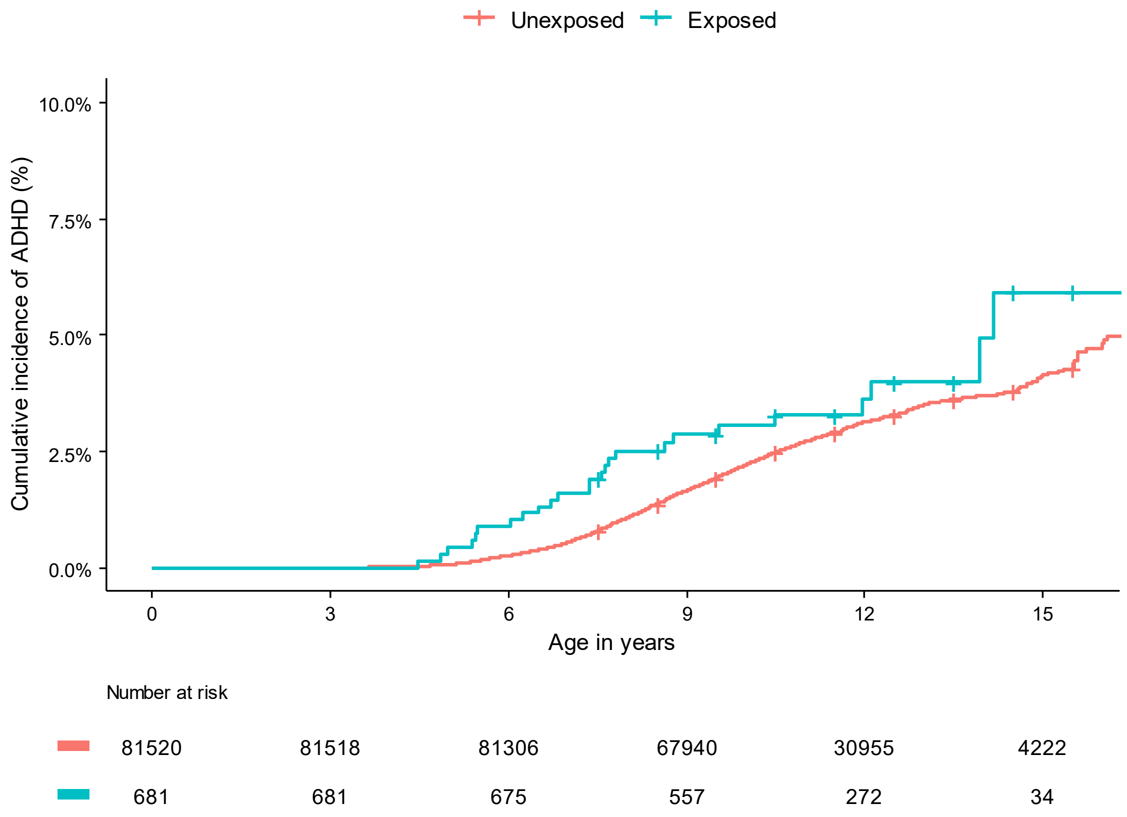

eFigure 6. Crude Kaplan-Meier failure curves showing cumulative incidence of ADHD in childhood among benzodiazepine and/or z-hypnotic exposed compared to unexposed.

Abbreviation: ADHD, Attention-Deficit/Hyperactivity Disorder.

© 2022 Sundbakk LM et al. *JAMA Network Open.*

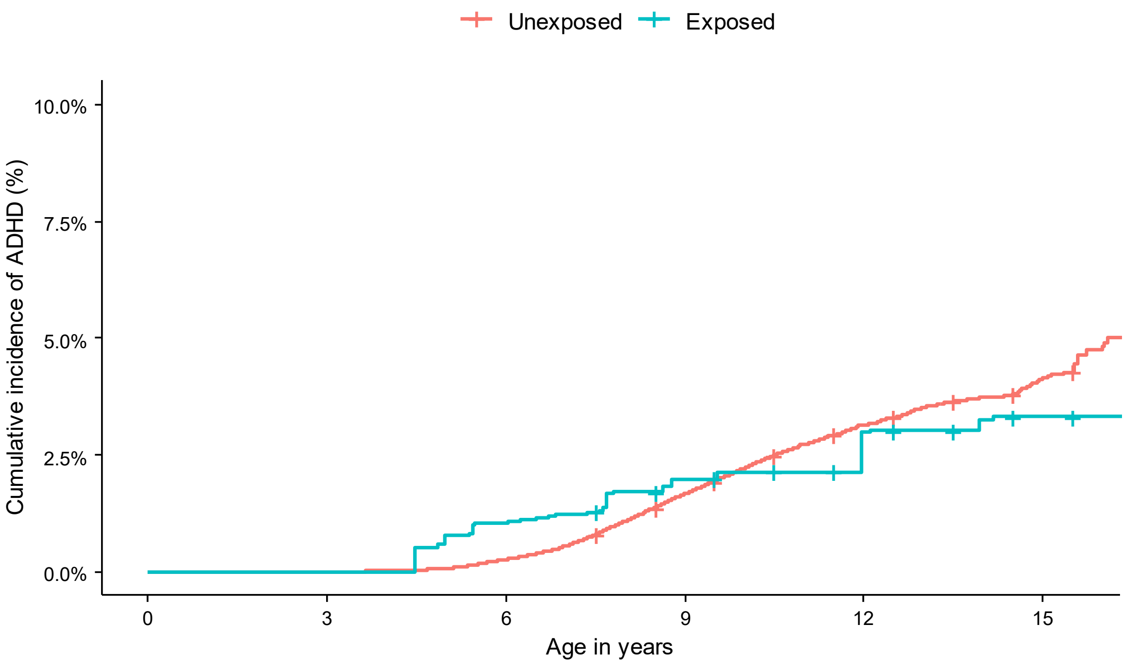

Number at risk

Unexposed	81543	81541	81328	67954	30955	4223
Exposed	617	617	611	508	235	23

eFigure 7. Weighted Kaplan-Meier failure curves showing cumulative incidence of ADHD in childhood among benzodiazepine and/or z-hypnotic exposed compared to unexposed.

Abbreviation: ADHD, Attention-Deficit/Hyperactivity Disorder.

© 2022 Sundbakk LM et al. *JAMA Network Open.*

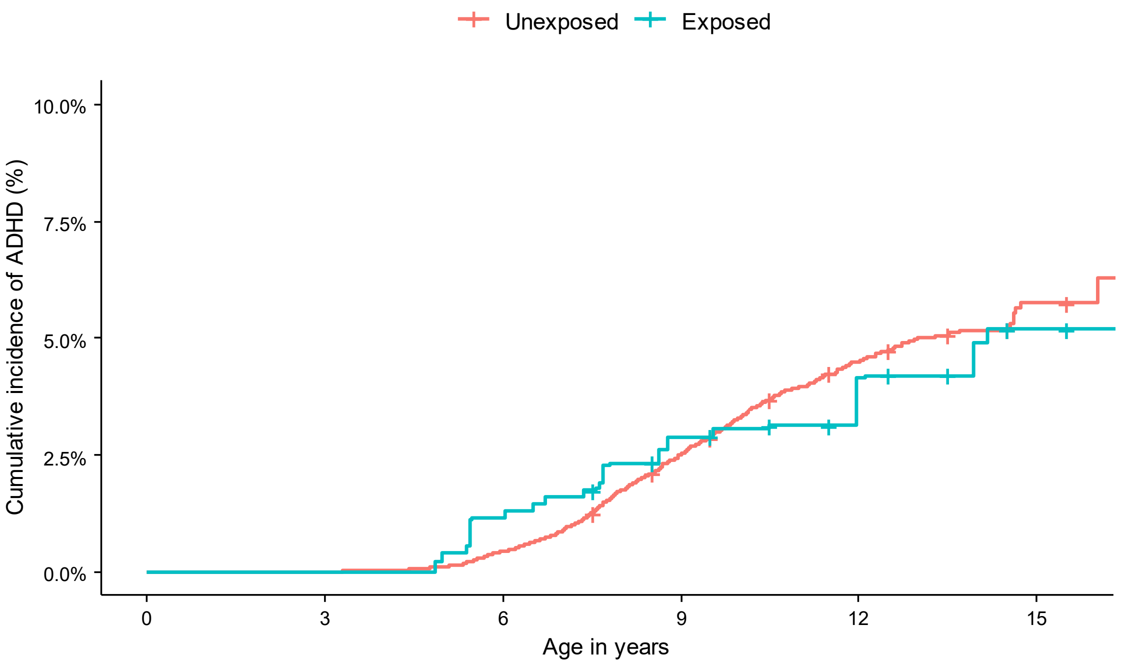

Number at risk

Unexposed	19124	19123	19040	15658	6716	850
Exposed	446	446	441	368	161	20

eFigure 8. Weighted Kaplan-Meier failure curves showing cumulative incidence of ADHD in childhood among benzodiazepine and/or z-hypnotic exposed compared to unexposed in the mental health sample.

Abbreviation: ADHD, Attention-Deficit/Hyperactivity Disorder.

© 2022 Sundbakk LM et al. *JAMA Network Open.*

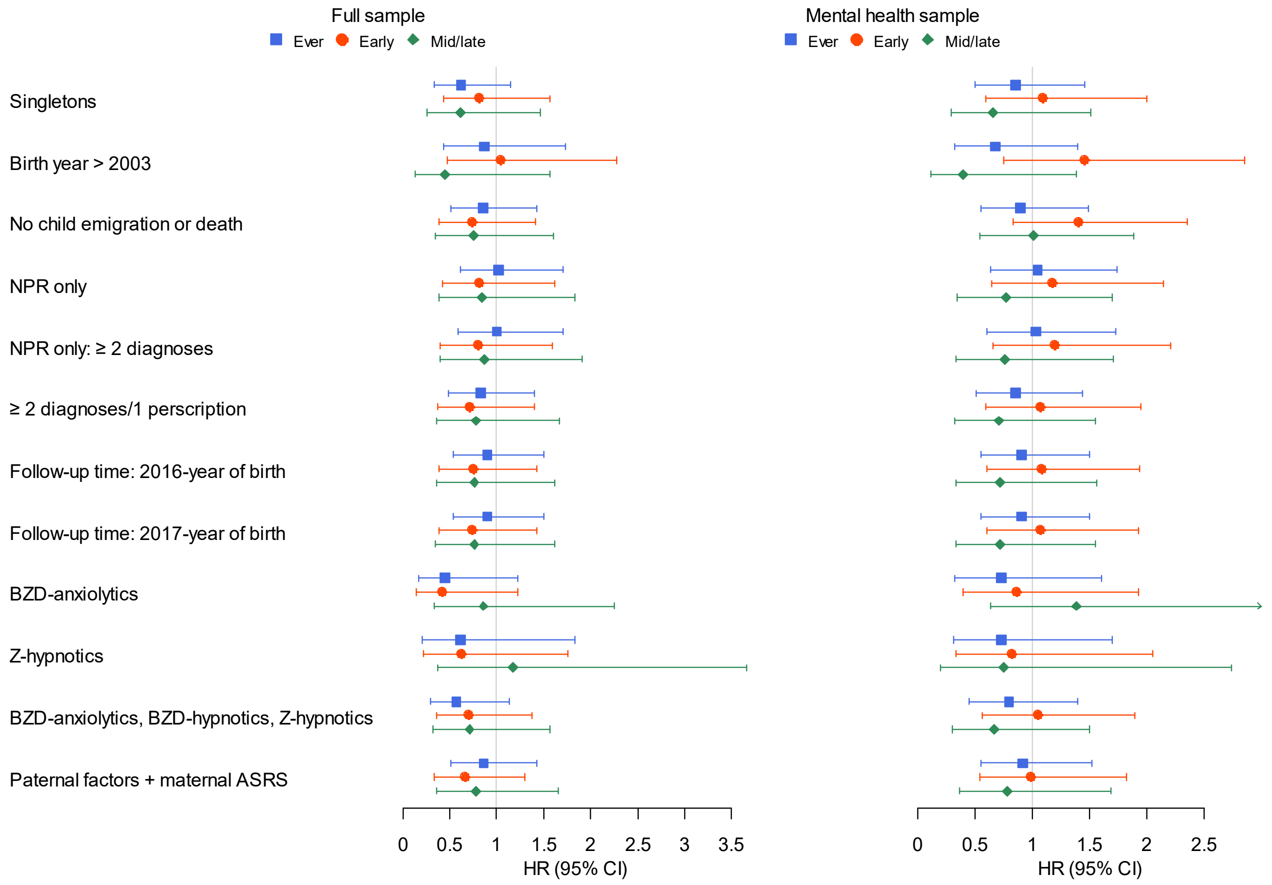

eFigure 9. Results from subgroup and sensitivity analyses. Abbreviations: ASRS, Adult ADHD Self-Report Scale; BZD, benzodiazepine; CI, confidence interval; HR, hazard ratio; NPR, Norwegian prescription registry.

© 2022 Sundbakk LM et al. *JAMA Network Open.*

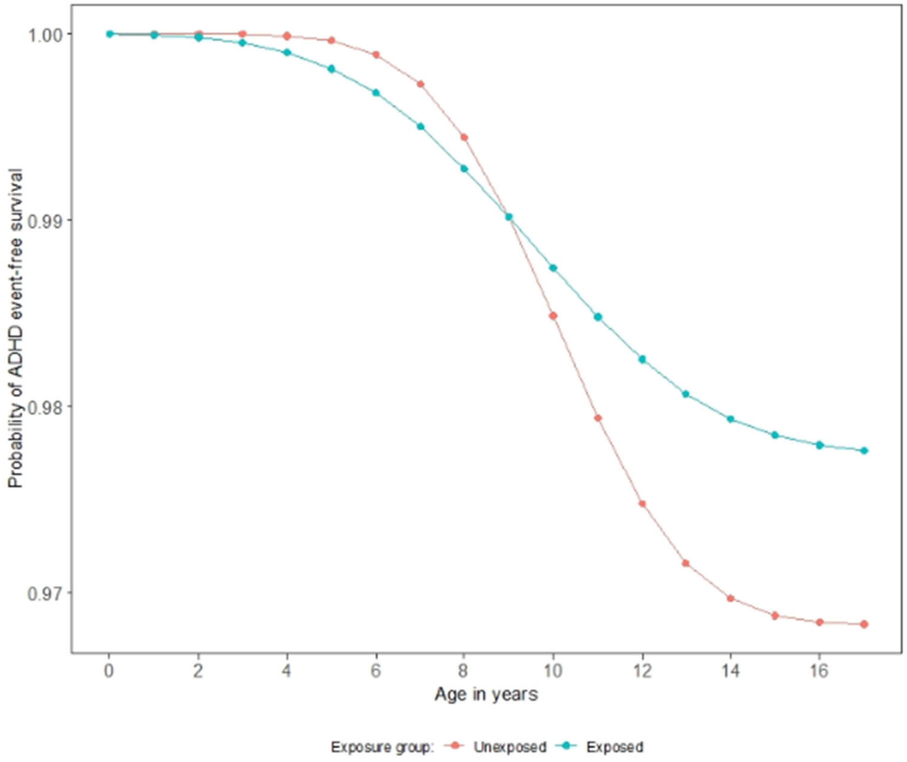

eFigure 10. Survival curves standardized for baseline covariates and baseline values of time-varying covariates.

Abbreviation: ADHD, attention-deficit/hyperactivity disorder.

© 2022 Sundbakk LM et al. *JAMA Network Open.*
