# Supplementary material for: Association of Prenatal Exposure to Benzodiazepines and Z-Hypnotics With Risk of Attention-Deficit/Hyperactivity Disorder in Childhood
Source: JAMA Netw Open. 2022 Dec 15;5(12):e2246889. doi: 10.1001/jamanetworkopen.2022.46889 (PMC9856385; doi:10.1001/jamanetworkopen.2022.46889)
Supplement: Supplement 2. — Data Sharing Statement [file jamanetwopen-e2246889-s002.pdf]

Data Sharing Statement

Sundbakk. Association of Prenatal Exposure to Benzodiazepines and Z-Hypnotics With Risk of Attention-Deficit/Hyperactivity Disorder in Childhood. *JAMA Netw Open*. Published December 15, 2022. doi:10.1001/jamanetworkopen.2022.46889

Data
Data available: No
